# Clinical and basic implications of dynamic T cell receptor clonotyping in hematopoietic cell transplantation

**DOI:** 10.1172/jci.insight.149080

**Published:** 2021-07-08

**Authors:** Simona Pagliuca, Carmelo Gurnari, Sanghee Hong, Ran Zhao, Sunisa Kongkiatkamon, Laila Terkawi, Misam Zawit, Yihong Guan, Hassan Awada, Ashwin Kishtagari, Cassandra M. Kerr, Thomas LaFramboise, Bhumika J. Patel, Babal K. Jha, Hetty E. Carraway, Valeria Visconte, Navneet S. Majhail, Betty K. Hamilton, Jaroslaw P. Maciejewski

**Affiliations:** 1 Translational Hematology and Oncology Research Program, Department of Hematology and Oncology, Cleveland Clinic, Cleveland, Ohio, USA.; 2 University of Paris, Paris, France.; 3 Department of Biomedicine and Prevention, School of Medicine and Surgery, University of Rome Tor Vergata, Rome, Italy.; 4 Blood and Marrow Transplant Program, Department of Hematology and Oncology and; 5 Department of Quantitative Health Sciences, Cleveland Clinic, Cleveland, Ohio, USA.; 6 Department of Genetics and Genome Sciences, School of Medicine, Case Western Reserve University, Cleveland, Ohio, USA.; 7 Leukemia and Myeloid Disorders Program, Department of Hematology and Oncology, Cleveland Clinic, Cleveland, Ohio, USA.

**Keywords:** Hematology, Immunology, Adaptive immunity, T cell receptor

## Abstract

TCR repertoire diversification constitutes a foundation for successful immune reconstitution after allogeneic hematopoietic cell transplantation (allo-HCT). Deep TCR Vβ sequencing of 135 serial specimens from a cohort of 35 allo-HCT recipients/donors was performed to dissect posttransplant TCR architecture and dynamics. Paired analysis of clonotypic repertoires showed a minimal overlap with donor expansions. Rarefied and hyperexpanded clonotypic patterns were hallmarks of T cell reconstitution and influenced clinical outcomes. Donor and pretransplant TCR diversity as well as divergence of class I human leukocyte antigen genotypes were major predictors of recipient TCR repertoire recovery. Complementary determining region 3–based specificity spectrum analysis indicated a predominant expansion of pathogen- and tumor-associated clonotypes in the late post–allo-HCT phase, while autoreactive clones were more expanded in the case of graft-versus-host disease occurrence. These findings shed light on post–allo-HCT adaptive immune reconstitution processes and possibly help in tracking alloreactive responses.

## Graphical abstract

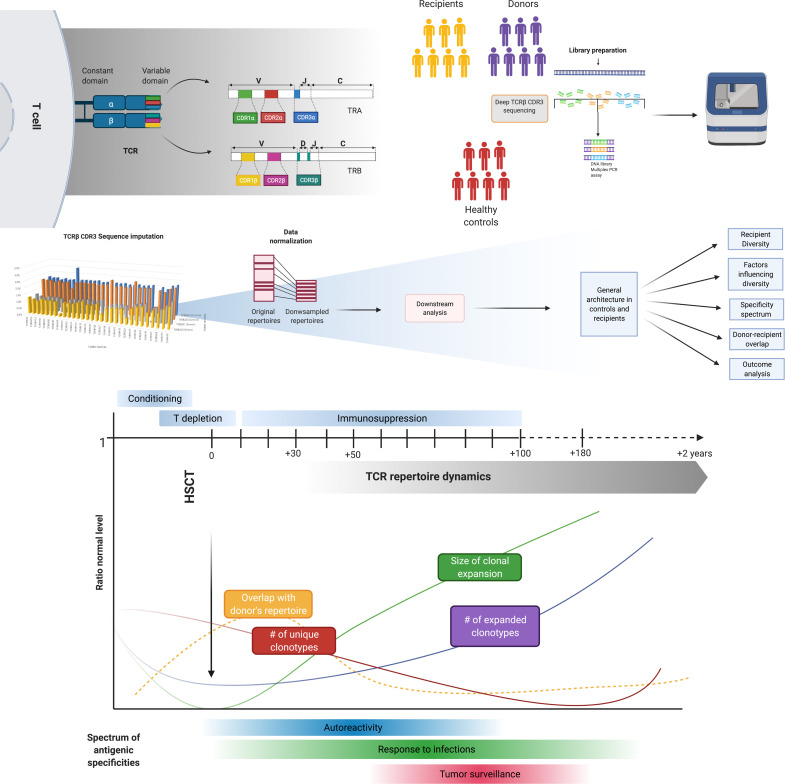

## Introduction

The success of allogeneic hematopoietic cell transplantation (allo-HCT) strictly depends on the ability of a donor’s engrafted immune system to both secure effective graft-versus-leukemia (GvL) responses and overcome infectious complications resulting from transplant-related immunodeficiency. While the spectrum of specificities recognized by the transplanted donor’s immune system constitutes a theoretical baseline, graft-versus-host disease (GVHD), infections, and tumor surveillance reactions affect the dynamics of recovery and direct a significant portion of the TCR repertoires toward antigenic triggers inherent to these processes (1, 2). Thus, the effectiveness of posttransplant immune reconstitution is highly dependent on both donor and recipient characteristics and is also influenced by the different transplant platforms (3). If the recovery of innate immunity is a relatively fast process, mainly occurring within the early posttransplant phase, the adaptive immune reconstitution requires the accomplishment of a cascade of events characterized by both thymus-independent expansion of T cells infused with the graft and thymus-dependent expansion of naive T cells derived from donor hematopoietic stem cells (4–8). Restoration and maintenance of a competent and sufficiently diverse TCR repertoire are of crucial importance for the immune balance among the processes of tolerance, response against pathogens, and GvL (9–11).

TCR diversity is the result of a stochastic process involving rearrangements of the V(D)J segments within the TRA, TRB, TRD, and TRG genes (12), which, along with junctional hypermutability, have a theoretical potential to generate up to 10^18^ complementary determining region (CDR) sequences (13). Within the αβ TCR repertoire, the CDR3 of the β chain (encoded by the TRB gene) is the principal site of antigen contact. CDR3 amino acid sequences can unambiguously mark TCRβ clonotypes as natural barcodes of individual T cell clones and, by extension, the recognized antigenic specificity (10). CDR3-encoded clonal specificities evolve de novo in the thymus or from the pool of clonotypes existent within the memory T cell compartment (12). These immune regenerative processes regress with age-related thymus involution, and in the allo-HCT setting, they can be impaired by the intensity of conditioning regimens, the prolonged immunosuppression, or the occurrence of GVHD (14).

Over the past 2 decades, several methods have been implemented for the characterization of the T cell recognition spectrum. Early investigations, exploiting spectratyping strategies, were consistent in showing the long-lasting skewing of post–allo-HCT TCR repertoires (11, 15). High-throughput sequencing of TCRβ chains now constitutes a more sensitive and accurate method to measure T cell diversity and thus T cell recognition repertoire after allo-HCT (16). Indeed, applications of this technology after allo-HCT demonstrated that delayed T cell recognition spectrum recovery and constrained TCR diversity correlate with worse outcomes, increased risks of infection, GVHD, and disease relapse (17–20). Recipient/donor age and cytomegalovirus (CMV) reactivation have been shown as factors influencing the skewing of recipients’ repertoire spectra (21). In addition, alloreactive processes may be indicated by the presence of hyperexpanded clones and restricted Vβ usage (18, 22).

Taking advantage of lessons learned from the previous attempts to recursively analyze T cell reconstitution dynamics after transplant (17, 18, 21, 23), we applied new bioanalytic strategies and clonotype reference data sets to study a prospective cohort of patients with myeloid neoplasia and their donors followed before and after allo-HCT. Our goal was to systematically, quantitatively, and qualitatively characterize the newly reconstituted recipient TCR β repertoires to understand how reciprocal patterns of donor/recipient clonal expansion and the evolution of recognition spectra may contribute to TCR diversification and divergence from donors’ repertoires.

## Results

### Landscape of TCR repertoires in healthy individuals versus recipients prior to HCT.

**Figure 1:**
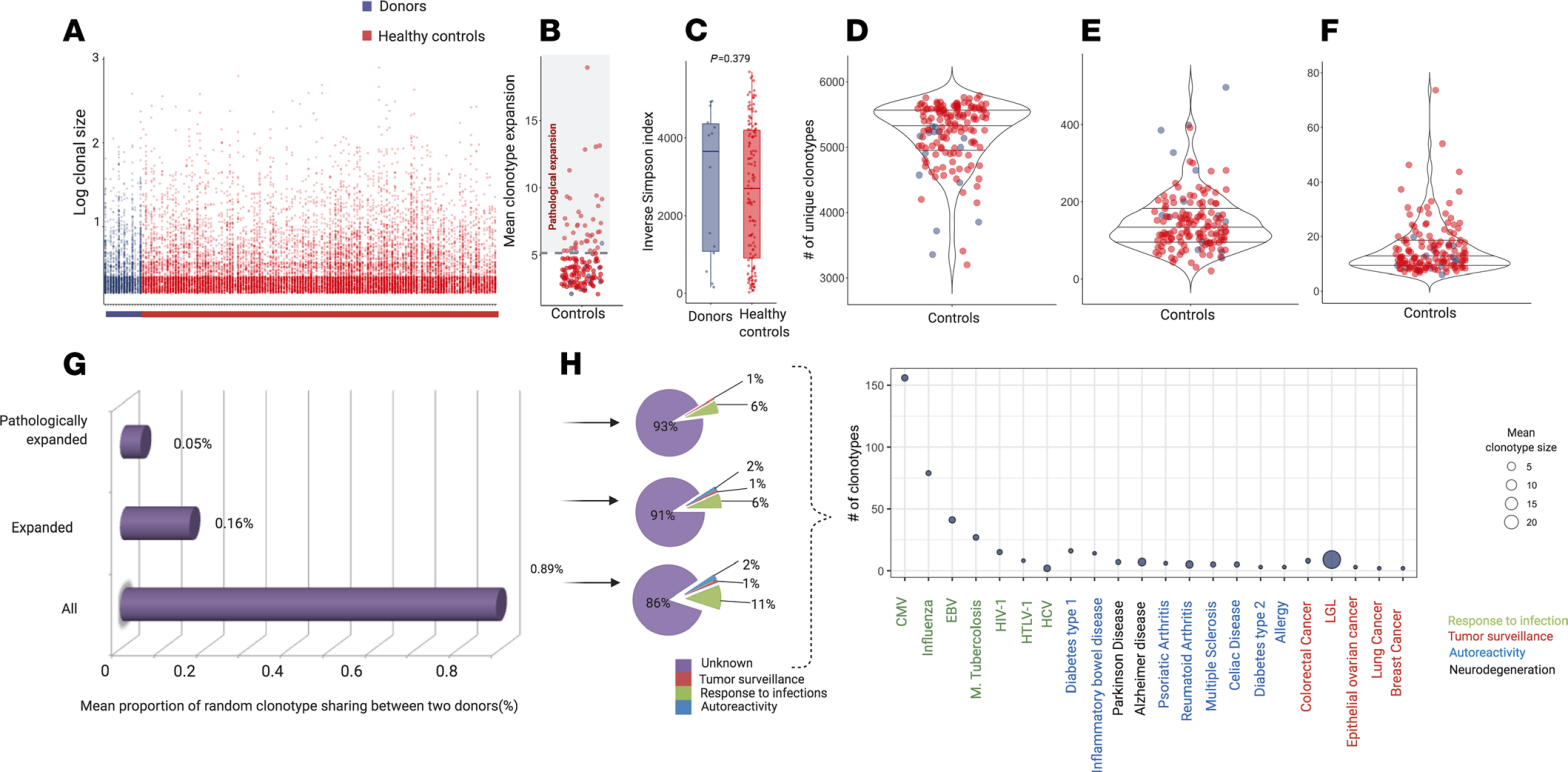
Structural organization of TCR repertoire in controls and patients before hematopoietic cell transplantation. (**A**) Clonotype size of healthy controls (HC, *n* = 131; red dots) and donors (*n* = 14; purple dots). Only expanded clonotypes are plotted (size greater than 1 template); *y* axis: size of each clonotype expressed in logarithmic scale (base 10); *x* axis: samples belonging to each group. Each dot represents a clonotype (rearrangement details in Supplemental Table 2A). (**B**) Mean size of all expanded clonotypes (size greater than 1 template), for HC (red dots) and donors (purple dots). Dot plot representing the mean size of expansion in 1 sample (donors: *n* = 14, HC: *n* *=* 131). The gray area configures the range of pathological expansion. Mean size of expansion: 4.72 (95% CI 4.31–5.12), gray dotted line: 5.12 (metrics details in Supplemental Table 2B). (**C**) ISI distribution in HC (*n* = 131) and donors (*n* = 14). Violin plots. Mean HC: 2488.94 (95% CI 2205–2772); Mean donors: 2826 (95% CI 1726–3926). Each dot represents the ISI in 1 sample (donors: *n* = 14, HC: *n* = 131). Wilcoxon rank sum test (*P* = 0.604). (**D**) Number of unique clonotypes in HC and donors (*n* = 145). Violin plots. Mean: 4886 (95% CI: 4812–4959). One dot per sample. (**E**) Number of unique expanded clonotypes in HC and donors (*n* = 145). Violin plot. Mean: 136 (95% CI: 124–148). One dot per sample. (**F**) Mean size of pathological expansion in HC and donors (*n* = 145). Violin plot. Mean: 15 (95% CI: 13–17). One dot per sample. (**G**) Proportion of repertoire overlapping between 2 healthy donors (Supplemental Table 3A). (**H**) Left: Pie charts representing the distribution of the known specificities of the clonotypes overlapping in donors in the three portions of the repertoire (whole, expanded and pathologically expanded). Right: Bubble chart depicting the number (y axis) and the mean size of expansion (bubble size) of overlapping clonotypes with known specificities (Supplemental Table 3B for rearrangement and annotation details). All of the analyses described here have been performed on the downsampled data set.

We prospectively collected 135 blood samples from 35 consecutive recipients and respective related donors (Table 1; Supplemental Table 1; and Supplemental Figure 1, A and B, for details on study design and patient characteristics; supplemental material available online with this article). To establish the normal distribution of the analytic TCR repertoire parameters, TCR repertoires from related donors and unrelated healthy individuals were used as benchmarks for normal and pathological parameters. Specifically, we quantitated the baseline characteristics of a healthy repertoire by building a data set of TCR rearrangements from 131 healthy controls (HCs) age matched with patients’ donors. To overcome the differences in sample depth, a common limitation of TCR repertoire analyses, we carried out a downsampling procedure of the original data set (see Methods and Supplemental Figure 2A) using the strategy of resampling donor/recipient and donor-matched HC TCR data sets to the lowest acceptable depth, determined to be 5420 CDR3 templates (Supplemental Table 1, parameter definitions). After the normalization process, we characterized the general landscape of a “normal” immune repertoire by analyzing rearrangement details of both donors and HCs (Supplemental Table 2, A and B; Figure 1, A–F; and Supplemental Figure 2). As a threshold of the pathological expansion, we arbitrarily used the upper limit of the 95% CI of the average size of expanded clonotypes in donors and HCs (mean size of expansion of all clonotypes of size > 1 template: Figure 1, A and B; 4.72 templates [95% CI 4.32–5.12]). A comparative analysis using the Inverse Simpson Index (ISI) (24, 25) showed that donor and HC CDR3 spectra did not differ, and thus they were combined wherever needed (Figure 1C). Controls displayed CDR3 diversity characterized by a high number of unique, unexpanded clonotypes (Figure 1, D–F). We then characterized the overlap of donors’ repertoires (Figure 1, G and H), calculating the basal number of public clonotypes that 2 unrelated healthy individuals could share. Given that this number is dependent on sample depth as with most metrics, we first computed the actual number of shared clonotypes for each pair of unrelated samples in the normalized data set. Then, based on the average of those results, we calculated the proportion of “public” clonotypes (Supplemental Table 3, A and B). This analysis was performed for all expanded and pathologically expanded clones (see Methods for details). The degree of general overlap between 2 unrelated individuals was less than 1% and specifically 0.9% for the clonotypes of all sizes, 0.16% for all expanded (size > 1 template), and 0.05% for all pathologically expanded clonotypes (size > 5 templates in our normalized cohort). We then annotated CDR3 sequences according to their specificity based on publicly available data sets (see Methods for details). Importantly, although most of the specificities remain unknown, a group of clonotypes whose spectra of reactivity were previously associated with infectious, cancer, and autoimmune processes could be identified throughout this analysis. In particular, across donor samples, the most represented CDR3 sequences were those related to response to CMV, influenza, and EBV infections. Curiously, the top expanded clonotype was one previously identified in large granular lymphocytic leukemia, a clonal lymphoproliferative disorder of cytotoxic T cells (Figure 1H and ref. 26).

We then compared the repertoires of donors and patients before transplant, following cytoreduction. Recipients’ cellular T cell systems were significantly compromised in terms of diversity spectrum (4382 vs. 4748 unique CDR3 tags, *P* = 0.035, Supplemental Figure 3A), with an increased mean size of pathological clonal expansions (21 vs. 14.6, *P* = 0.001), accounting for a generalized contraction of the TCR repertoire (Supplemental Figure 3, A–C). Similar conclusions arose when comparing the ISI of donors and recipients pretransplant (Supplemental Figure 3D) and when evaluating the changes of pathologically expanded ranks compared with donor frequencies (Supplemental Figure 3E).

### TCR diversity and its dynamics in recipients.

**Figure 2:**
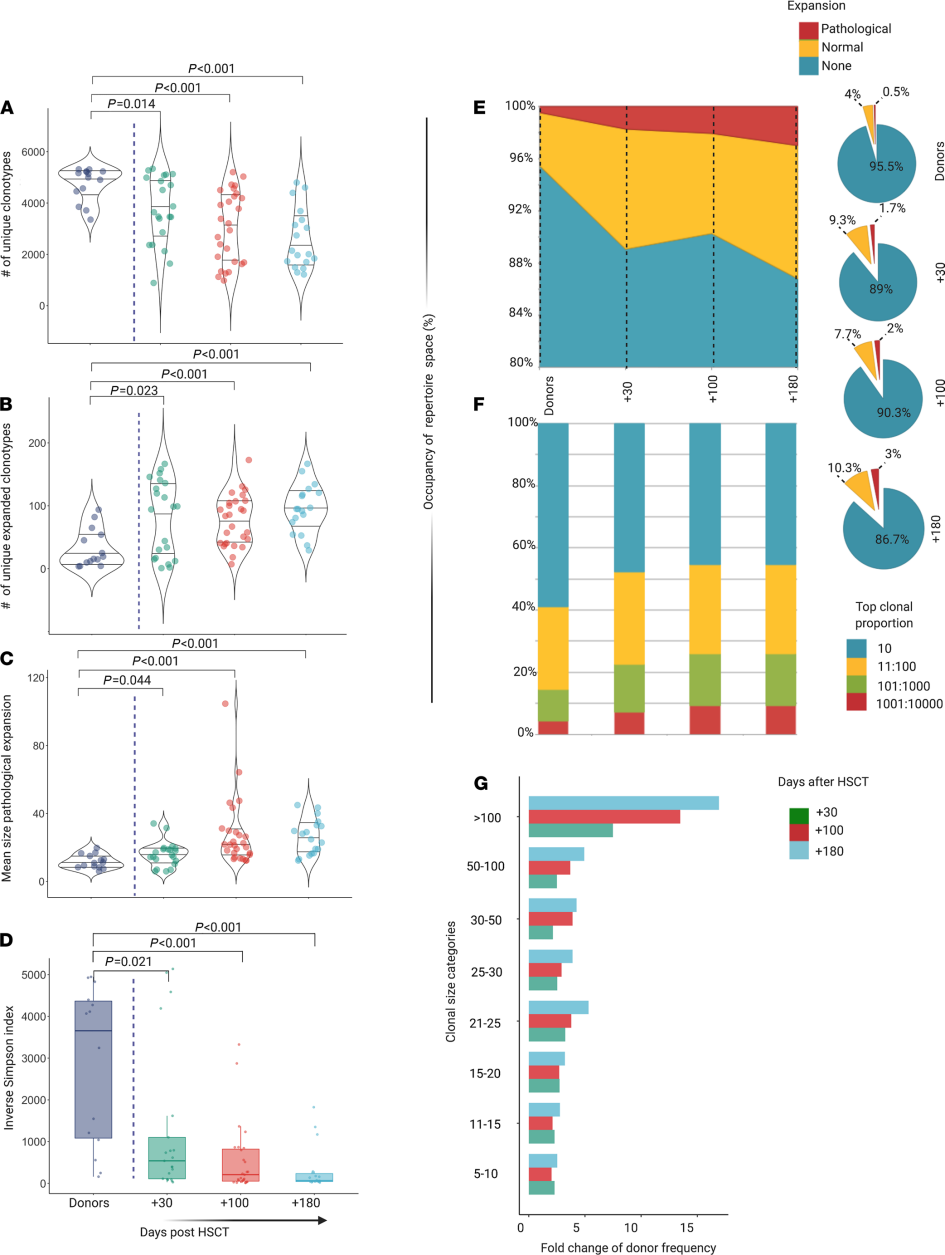
Diversity and structure of TCR repertoire in recipients after HCT. (**A**) Number of unique clonotypes in recipients at day +30 (*n* = 21), +100 (*n* = 27), and +180 (*n* = 18), compared with donors (*n* = 14) (Supplemental Table 4B). Violin plots. One dot per sample. (**B**) Number of unique expanded clonotypes in recipients at day +30, +100, and +180, compared with donors (Supplemental Table 4B). Violin plots. One dot per sample. Pairwise comparisons with donor group. Wilcoxon rank sum test (*P* = 0.014, *P* < 0.001, *P* < 0.001, respectively). (**C**) Mean size of pathological expansion (indicated as mean clonotype expansion-number of template-per clonotype-per sample) +30, +100, and +180, compared with donors (Supplemental Table 4V). Violin plots. One dot per sample. Pairwise comparisons with donor group. Wilcoxon rank sum test (*P* = 0.044, *P* < 0.001, *P* < 0.001, respectively). (**D**) ISI distribution in recipients at day +30, +100, and +180, compared with donors (Supplemental Table 4B). Box plots. One dot per sample. Pairwise comparisons with donor group. Wilcoxon rank sum test (*P* = 0.021, *P* < 0.001, *P* < 0.001, respectively). Purple, donors; green, day +30; red, day +100; light blue, day +180. (**E**) Distribution of the proportions of nonexpanded, normally expanded, and pathologically expanded specificities (filled area chart and pie charts, Supplemental Table 4D). (**F**) Distribution of clonal proportions. The bar graph depicts the distributions of the most expanded (top 10) to less expanded clonotypes (top 1001:10000). (**G**) Average number per sample of pathologically expanded clonotypes according to clonal size category in day +10 (green), +100 (red), and +180 (light blue), expressed as fold change compared with donor group (Supplemental Table 4C).

We then assessed post–allo-HCT metrics in recipients of the corresponding hematopoietic cell grafts at serial time intervals following transplantation (Supplemental Table 4, A–F). Globally, the diversity of TCR repertoires in recipients was significantly decreased after allo-HCT due to a decrease in the number of unique clonotypes (Figure 2A) and an increase in the number and size of expanded clones (Figure 2, B and C). As expected, the ISI followed this trend (Figure 2D). Although most of the repertoire space was occupied by nonexpanded or normally expanded clonotypes, the pathologically expanded counterpart increased progressively (2.1%, 2.5%, and 3.7%; at +30, +100, and +180 days, respectively; Figure 2, E–G), contributing to the reduced diversity of the repertoire in the latest posttransplant phases.

**Figure 3:**
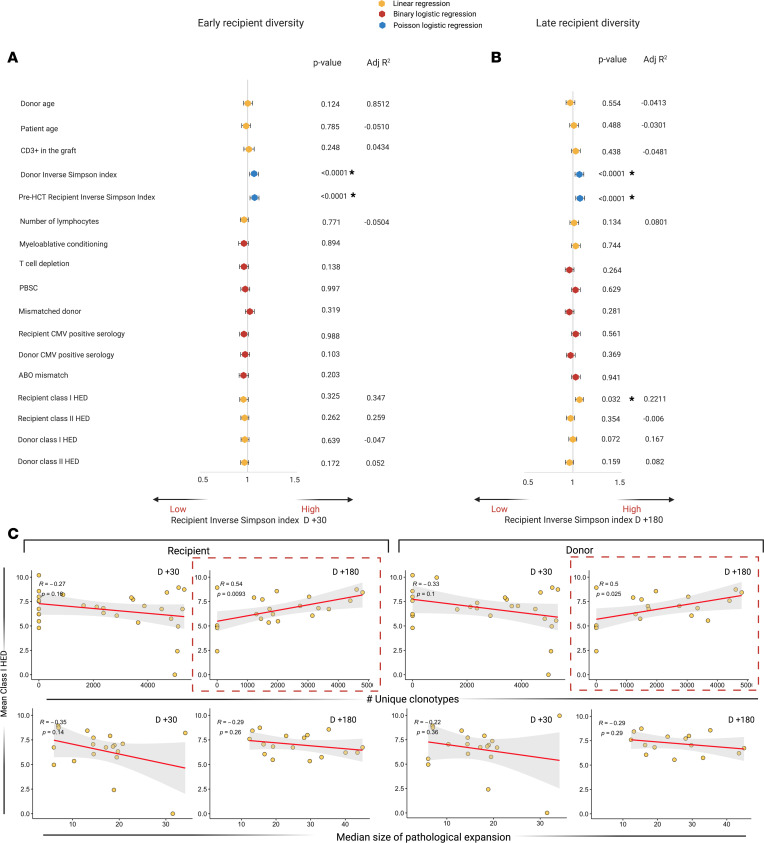
Factors influencing early and late posttransplant TCR diversity. (**A**) Univariable analysis of factors influencing early repertoire diversity. Forest plot indicating the odds ratio and the 95% CIs of the impact of several continuous or binary predictors on diversity (ISI day +30, considered as continuous variable). Univariable models are built either as a linear regression or logistic regression (binomial or Poisson regression as per legend). (**B**) Univariable analysis of factors influencing late repertoire diversity (ISI at day +180, modeling as in **A**). (**C**) Linear regressions showing the correlations between recipient (left panels) and donor (right panels) mean class I HED and diversity parameters: *x* axes represent the number of unique clonotypes (upper panels), and median size of pathological expansion (indicating the median number of templates per pathologically expanded clonotypes, lower panels).

We found that donor and recipient pre-HCT ISIs were predictive of a broader TCR repertoire diversity in both early and late time points post-HCT (*P* < 0.0001), while T cell content in the graft, intensity of conditioning regimen, graft source, T cell depletion, type of donor, and donor/recipient CMV status did not affect any of those parameters (Figure 3, A and B).

We then studied the impact of donor and recipient structural divergence of HLA molecules (HLA evolutionary divergence, HED), as an indirect measure of the extent of immunopeptidome presented and, hence, as an immunogenetic predictor possibly influencing post–allo-HCT repertoire diversification. We found that recipient mean class I HED impacted day +180 ISI (*P* = 0.032) as a result of a direct correlation with a higher number of unique clonotypes (R = 0.64, *P* = 0.009). Donor class I HED was also directly correlated with late TCR richness (R = 0.5, *P* = 0.025), although no impact was seen on the resulting ISI (Figure 3, B and C).

### Similarity of TCR repertoires in recipients.

**Figure 4:**
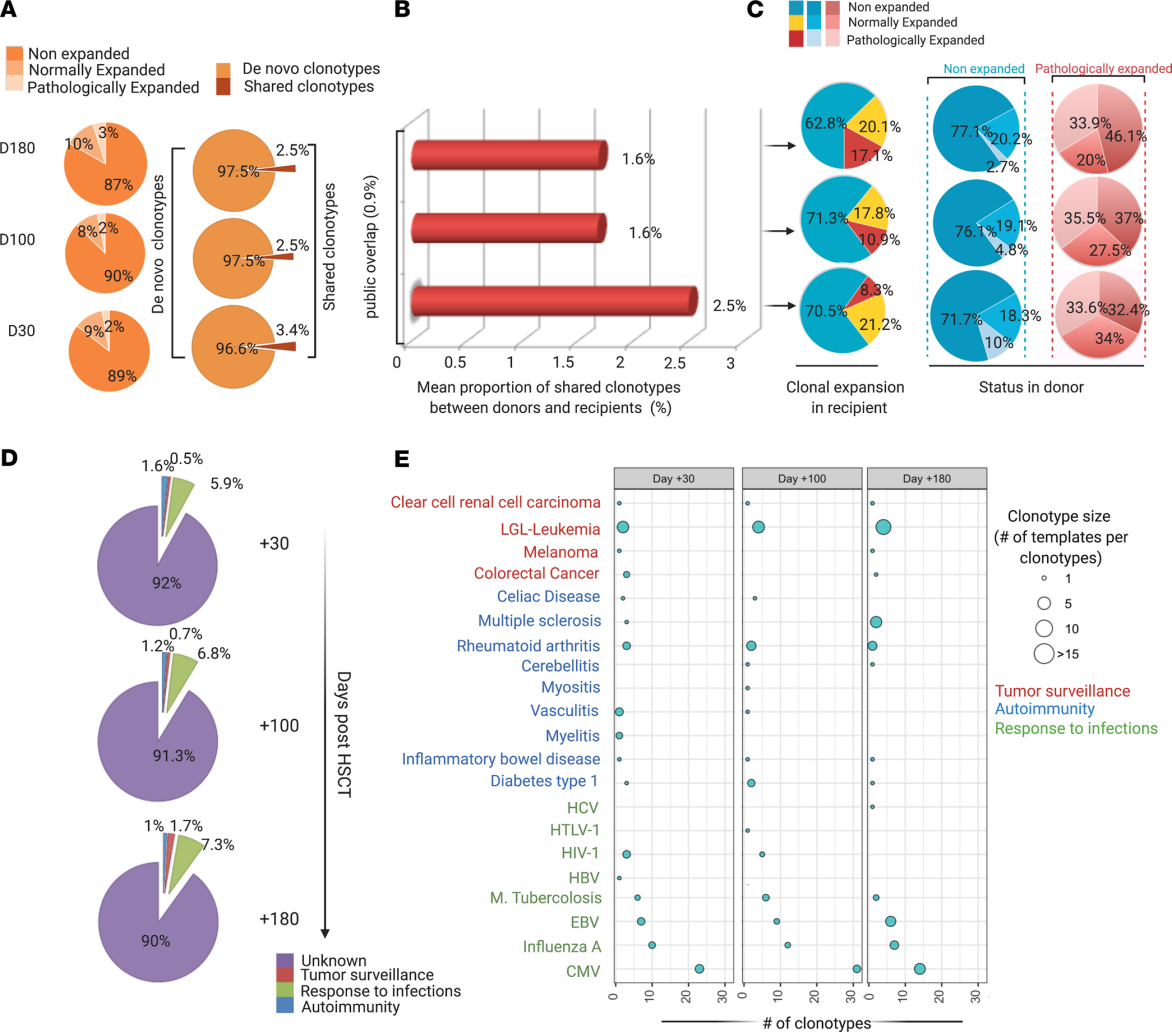
Donor-recipient overlap analysis. (**A**) Proportion of de novo and overlapping specificities at day +30, +100, and +180. Rearrangement details and number of donor-recipient shared clonotypes are reported in Supplemental Table 5, A and B. (**B**) Proportion of donor-recipient shared specificities after subtraction of public interindividual overlap (0.9% of repertoire). (**C**) Origin of shared clonotypes according to clonal expansion in recipients and donors (Supplemental Table 5C). The first set of pie charts (blue for nonexpanded, yellow for normal expanded, red for pathologically expanded) refers to the status of clonal expansion in recipients at the 3 time points. The second and third set of pie charts refer instead to the proportion of donor repertoires at the origin of nonexpanded (blue) and pathologically expanded (red) clonotypic fractions in recipients. (**D**) Distribution of the known specificities of donor-recipient overlapping clonotypes in posttransplant groups. (**E**) Bubble chart depicting the number (*x* axis) and the mean size of expansion (bubble size) of donor-recipient overlapping clonotypes with known specificities (Supplemental Table 5A for rearrangement and annotation details). Of note only a selection of most represented disease/pathogen associated groups is captured in the figure for visualization purposes. All of the analyses described here have been performed on the downsampled data set.

We performed a qualitative analysis of the specificity spectrum shared between donors and recipients in the posttransplant course. Specifically, we calculated the number of overlapping CDR3 sequences for each recipient-donor sample pair and computed the overlap coefficient (see definitions in Methods and Supplemental Table 1), a metrics enabling consideration of both sample depth and the relative abundance of each shared clonotype. Furthermore, to overcome the problem related to the presence of public clonotypes possibly affecting this analysis, we deducted the average overlap calculated by combinatorial pairwise computation of all the clones shared between 2 donors (see Methods, Supplemental Table 3, and Figure 3, A and B). Using this strategy, we demonstrated that only a small portion of recipients’ TCR repertoires were shared with the respective donors across the whole posttransplant period. Without considering the degree of overlap found in the general population, this similarity was higher in the first month but diminished later (from 2.5% to 1.6%), while more than 97% of the repertoire was represented by de novo clonotypes, at a given depth (Figure 4, A and B). When we tried to dissect the composition of the shared repertoire, most of the nonexpanded CDR3 sequences in recipients were found to derive from nonexpanded donors’ clones while a conspicuous part of pathologically expanded clonotypes in patients were also pathologically expanded in donors (Figure 4C). Consistent with this finding, we could demonstrate a direct correlation of the size of clonotype expansion between recipients and donors (Supplemental Figure 4A). Nevertheless, pairwise recipient-donor overlap coefficients remained constantly low across the various time points, possibly indicating that no further donor-derived T cell expansion occurred along the latest posttransplant phase (Supplemental Figure 4B).

We then investigated the dynamics of known donor-recipient shared clonotypes, represented by overlapping CDR3 sequences. To do so, we used a reference data set that we generated aggregating all the known immune target/disease associated clonotypes (see Methods). Indeed, known specificities accounted for only 8%–10% of recipient TCR Vβ repertoires. Known clonotypes were grouped according to a *per pathology* classification (e.g., autoimmune-, allergy-, cancer-, and pathogen-associated epitopes). More specifically, shared CDR3 sequences recognizing epitopes from CMV, EBV, and influenza A infections were the most represented in the recipient cohort across the whole posttransplant period. In contrast, autoimmune-associated specificities were the most expanded, especially in the first phase after HCT, while clonotypes related to antitumor adaptive immune responses were mostly identified in the latest phases (Figure 4D). Of note, selected specificities are shown in Figure 4E.

### Tracking the specificity of immune responses in posttransplant repertoires.

In an effort to track specific immune responses, we characterized the proportion and the clonal expansion of all the clonotypes with recognizable specificities present in our samples. Interestingly, we found different patterns of expansion across the post–allo-HCT period. Specifically, while clones associated with response to infection and tumor surveillance were more expanded in late posttransplant phases, somehow measuring the antigenic challenges of a more immunocompetent adaptive system, autoreactive clonotypes were instead inflated at day +30 and particularly expanded at GVHD onset (Supplemental Figure 5).

### Clinical correlations including GVHD and CMV infections.

Univariable Cox regression analysis was performed to assess how diversity (ISI) and donor-recipient overlap (overlap coefficient), along with the other quantitative metrics (number of unique clonotypes and of pathologically expanded clonotypes as well as median size of pathological expansion in recipients at day +30, +100, and +180) influenced the probability of survival, acute and chronic GVHD, and relapse (see Table 2). Overall, a higher number of unique clonotypes correlated with better survival outcomes (HR 0.99 [95% CI 0.998–0.99] *P* = 0.043) and lower risk of relapse (HR 0.98 [95% CI 0.97–0.99], *P* = 0.005) while the presence of a higher proportion of pathological expansions negatively affected OS (HR: 1.04 [95% CI 1.00–1.09], *P* = 0.047). Moreover, a higher overlap at day +30 was associated with an increased risk of chronic GVHD (HR 1.02 [95% CI 1–1.04], *P* = 0.023), implying a possible role of donor-imprinted specificities in fostering this complication.

**Figure 5:**
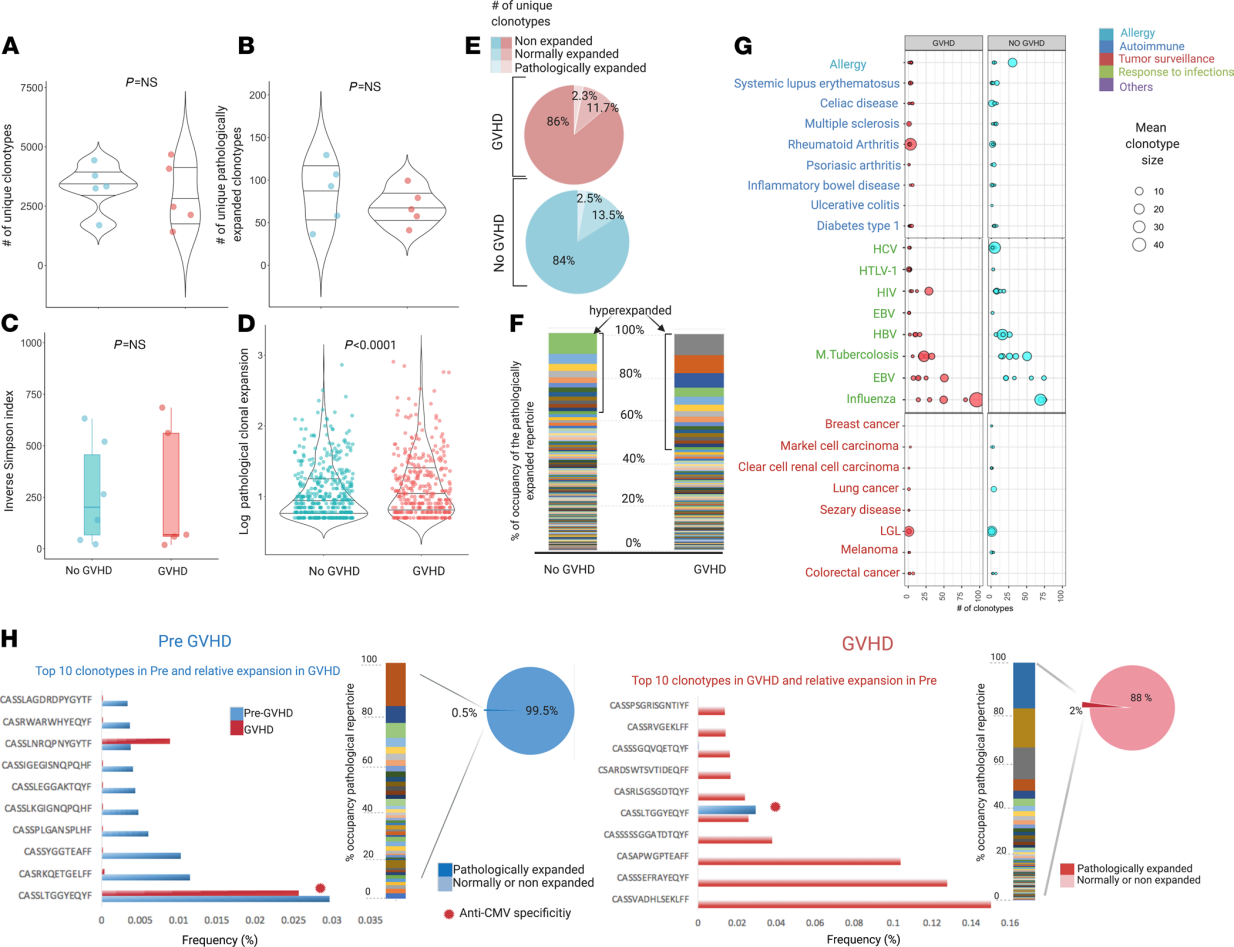
Structure of TCR repertoire at acute GVHD onset. (**A**) Number of unique clonotypes at onset of acute GVHD (*n* = 5, red) and matched patients never developing a GVHD (*n* = 5). Violin plot. One dot per sample. Wilcoxon rank sum test (*P* = 0.814). (**B**) Number of unique pathologically expanded clonotypes in GVHD and non-GVHD groups. Violin plot. One dot per sample. Wilcoxon rank sum test (*P* = 0.690). (**C**) ISI distribution in GVHD and non-GVHD groups. Box plots depicting median and IQR. One dot per sample. Wilcoxon rank sum test (*P* = 0.792). (**D**) Size of pathologically expanded clonotypes in GVHD and non-GVHD groups. Violin plot: *y* axis expressed in log_10_ scale. One dot per clonotype. Wilcoxon rank sum test (*P* = 0.00096). (**E**) Proportion of nonexpanded, normally expanded, and pathologically expanded specificities. (**F**) Structural organization of the pathologically expanded portion of the repertoire. Bar plot representing each unique clonotype (each color) with its clonal size (area occupied by each color); *y* axis: proportion of occupancy of the pathologically expanded clonal space. The hyperexpanded group belong to all the clonotypes of size larger than 100. (**G**) Bubble chart illustrating the number (*x* axis) and mean size of expansion (bubble size) of clonotypes with known specificities in GVHD (red) and non-GVHD (light blue) groups (Supplemental Table 6A for rearrangement and annotation details). Of note, a selection of most represented disease-pathogen associated groups is captured. (**H**) Longitudinal analysis of 1 index case (CCF43) with clonotype analysis of pre-GVHD (day +30) and GVHD sample (day +90). Multicolor bars indicate proportion of occupancy of pathological clonal space for each unique clonotype (each color) and respective clonal size (bar area). Note: To capture all specificities in samples in the study analysis, in **H** the analysis was performed on the nondownsampled data set, with specificity frequency calculated as (# templates of each unique clonotype [clonal size]/total depth [total # templates]) × 100. Total depth: 24339 pre-GVHD, 70832 GVHD (Supplemental Table 6C).

Our group and others showed that the presence of immuno-dominant CD8^+^ clonotypes may be identified as mono- or oligo-expansion of specific Vβ families in blood samples and tissues from patients developing acute GVHD (22). We therefore explored the characteristics and the polarization of TCR expansion in patients experiencing this complication. Samples were studied in 5 patients at acute GVHD onset and compared with those obtained from patients who had never experienced either acute or chronic GVHD. Quantitative analysis showed no difference in terms of diversity and number of pathologically expanded clonotypes between the 2 groups (Figure 5, A–C). However, a dissimilar composition of the pathological expanded fraction was observed (Figure 5D). Specifically, although only around 2% of unique clonotypes were pathologically expanded in both subgroups (Figure 5E), hyperexpanded fraction was increased in patients with acute GVHD (Figure 5F). When the known specificities were analyzed, no differences were seen in terms of distribution and size of pathogen-associated and autoimmune-associated clonotypes between the 2 groups (Figure 5G). Longitudinal analysis of 1 index case with grade II intestinal and cutaneous acute GVHD at day +90 showed a higher number of hyperexpanded clones and a greater median size of pathological expansions as compared with those found in the sample collected at day +30 when no GVHD was present (Figure 5H). Similarly, the distribution and composition of hyperexpanded clonotypes were different between the 2 samples collected before and at the onset of GVHD (Figure 5H and Supplemental Figure 6).

**Figure 6:**
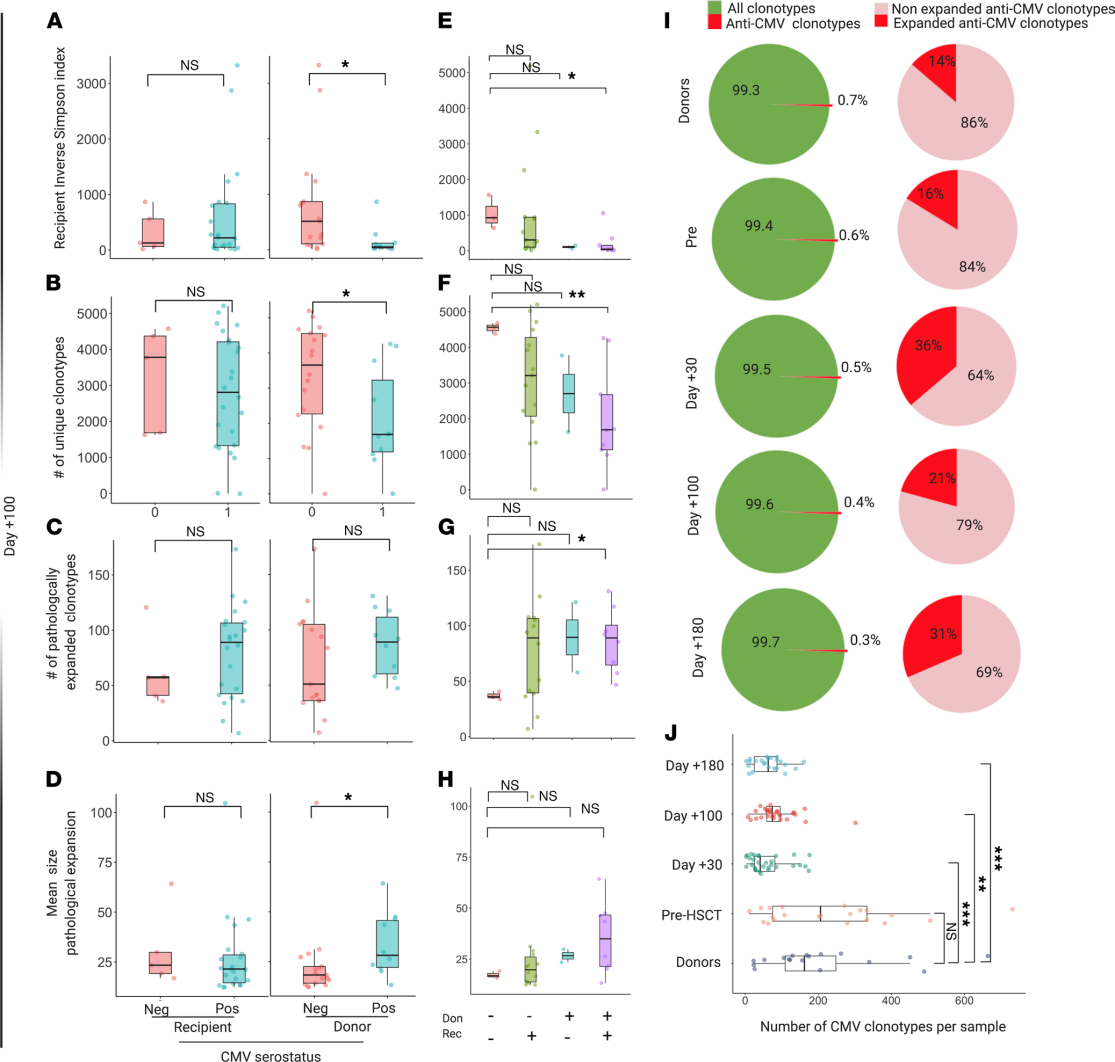
Impact of CMV on TCR organization. (**A**) ISI distribution in recipients at day +100 according to recipient (left) and donor (right) CMV serological status. Recipient negative CMV status (pink box, *n* = 5); recipient positive CMV (light blue, *n* = 22); donor negative CMV (pink, *n* = 17); donor positive CMV (light blue, *n* = 10). One dot per sample. Recipient status: *P* = 0.832; donor status: *P* = 0.011. (**B**) Number of unique clonotypes in recipients at day +100 according to recipient (left) and donor (right) status. Recipient: *P* = 0.662; donor: *P* = 0.028. (**C**) Number of unique pathologically expanded clonotypes in recipients at day +100 according to recipient (left) and donor (right) status. Recipient: *P* = 0.512; donor: *P* = 0.183. (**D**) Mean size pathological expansion in recipients at day +100 according to recipient (left) and donor (right) CMV serological status. (**E**) ISI distribution in recipients at day +100 according to D/R CMV status match. D–/R– (*n* = 3); D–/R+ (*n* = 14); D+/R– (*n* = 2); D+R+ (*n* = 8). Pairwise comparisons with D–/R– category. (D–/R+: *P* *=* 0.244; D–/R+: *P* = 0.210; D+/R+: *P* *=* 0.024.) (**F**) Number of unique clonotypes in recipients at day +100 according to D/R CMV status match. Pairwise comparisons with D–/R– category. (D–/R+: *P* = 0.164; D+/R–: *P* = 0.200; D+/R+: *P* = 0.0091.) (**G**) Number of unique pathologically expanded clonotypes in recipients at day +100 according to D/R CMV status match. Pairwise comparisons with D–/R– category. (D–/R+: *P* = 0.147; D+/R–: *P* = 0.600; D+/R+: *P* *=* 0.012.) (**H**) Mean size pathological expansion in recipients at day +100 according to D/R CMV status match. Pairwise comparisons with D–/R– category. (D–/R+: *P* = 0.768; D+/R–: *P* = 0.267; D+/R+: *P* = 0.084.) (**I**) Proportion of anti-CMV specificities within the whole repertoire. (**J**) Number of anti-CMV clonotypes per sample for each sample group. Pairwise comparisons with donor category. (Pre-HCT *P* = 0.703; day +30 *P* = 0.00079; day +100 *P* = 0.00687; day +180 *P* = 0.00061.) All analyses included in the study of anti-CMV CDR3 sequences were performed on the nondownsampled data set to capture all specificities. All *P* values were 2 sided. Wilcoxon rank sum. *P < 0.05, **P < 0.01, ***P < 0.001.

To explore how CMV serostatus and infections may shape posttransplant TCR architecture, we performed a deep quantitative cross-sectional analysis and studied dynamic changes of known anti-CMV specificities. This analysis was specifically carried out on the non-normalized data set in order to capture all the clonotypes across the specimens in the study. Interestingly, while the recipient’s CMV seropositivity status did not influence diversity metrics at day +100 (Figure 6, A-D), the donor’s positive serostatus was associated with a lower diversity in recipients (*P* = 0.011, lower number of unique clonotypes [*P* = 0.028] and a higher mean of pathological expansion [*P* = 0.031]; Figure 6, A–D). Specifically, when we studied donor/recipient serologic configurations, this difference was evident between double-negative (D–/R–) and double-positive (D+/R+) CMV subgroups (Figure 6, E–H). Overall, each sample contained an average number of 118 unique CMV-associated specificities. The number of those clonotypes at day +100 was significantly lower in all posttransplant samples compared with donors or pre-HCT specimens (Figure 6, I and J), possibly as a consequence of the dramatic decrease of post–allo-HCT diversity. Of note, although the distribution of those specificities was similar across recipient and donor groups based on CMV serological status (Supplemental Figure 7A), patients receiving a graft from CMV-negative donors had a significantly higher anti-CMV TCR expansion (Supplemental Figure 7B). Also, no difference in distribution of CMV-associated clonotypes at day +100 was seen in patients developing CMV infection/reactivation (Supplemental Figure 7C). As an example, the impact of global diversity and the kinetics of CMV-associated specificities in case of CMV reactivation is studied in an index case represented by a recipient diagnosed with AML and receiving a bone marrow transplant from an HLA-matched unrelated donor (CMV status: D–/R+) (Supplemental Figure 7D).

## Discussion

A delayed and/or functionally impaired T cell immune reconstitution is a crucial determinant of clinical outcomes following HCT (10, 27). Despite the progress achieved in the last decades in deciphering the dynamics of T cell reorganization after HCT (17, 11, 28), critical aspects of TCR repertoire recovery have not been sufficiently clarified due to the complexity of the clinical course, the logistics needed to capture clinical events and samples in a systematic fashion, and the technological limitations of bioanalytic pipelines. To this end, deep next-generation sequencing (NGS) has rendered TCR CDR3 molecular characterization a powerful tool to study adaptive immune systems. The capability of this technology can be fully exploited in the HCT setting in order to analyze the quantitative aspects of TCR repertoires as well as to study shared and new specificities and identify prevailing antigenic drivers.

Here, we present the results of a comprehensive analysis of the changes in TCR repertoires in a homogenous cohort of patients receiving allo-HCT using deep TCR Vβ CDR3 high-throughput sequencing. We devised TCR repertoire metrics capturing all components of diversity and similarity. To overcome the problems related to the absence of references for the study population, we constructed a “control” TCR repertoire from a large cohort of age-matched HCs that were combined with our donor data set in order to establish ranges for all the parameters of the study, including intrinsic diversity, patterns of clonal expansion, and public overlap. We then investigated how those aspects influenced posttransplant outcomes across different allo-HCT platforms in a cohort of patients with myeloid malignancies. Moreover, we deployed innovative bioanalytic pipelines to explore the target spectrum of TCR specificities.

With the limitations inherent in the tremendous complexity of transplant platforms and the diversity of clinical scenarios (e.g., different types of disease, conditioning regimens, GVHD prophylaxis, etc.), previous studies have indicated that patients’ TCR repertoires are severely altered in terms of diversity and skewing in relationship to clinical outcomes and D/R clonotype sharing (11, 28, 21, 23). Building on these results, our study has investigated the diversity parameters systematically and rationally by examining the configuration of the TCR spectrum in all its dimensions. Using potentially novel implemented pipelines, we also studied clonotypic interactions with donors and imputed specificity spectra of the newly generated repertoires. We demonstrate that the hallmarks of the newly generated TCR repertoires include a) progressive reduction of the number of unique clonotypes (likely related to lymphodepletion and immunosuppression), b) an overall increase in the number of pathologically expanded clonotypes, and c) a parallel rise of clones with defined specificities along the posttransplantation period. Within these dynamics, decreased TCR variability was a general feature of all types of transplant platforms whereby nuances were dictated by donor and recipient pretransplant TCR repertoire diversity and genotypic HLA configurations. The latter observation confirms the role of highly divergent HLA genotypes in eliciting diverse T cell specificities, also in the post–allo-HCT setting (29). Per extension, this aspect is in line with previous work showing how D/R HLA genotypes may shape the recipient minor histocompatibility immune peptidome responsible for the direction and the intensity of alloreactive responses (30).

In the case of acute GVHD, both in a cross-sectional and serial analysis, we showed that an increased size of a few clonotypes, likely associated with alloreactive responses, accounted for the majority of the expanded clonal spectrum, thus diminishing the overall diversity of the TCR repertoire and impairing competent immune surveillance. Consistent with this conclusion, patients with higher TCR diversity had better survival outcomes and a lower risk of relapse, while a higher median size of pathological expansion and an increased overlap with donor repertoires was associated with risk of chronic GVHD.

Based on the theory that CDR3 clonotypes can serve as markers of specific antigenic drives, we have also performed a comprehensive analysis of the spectrum of TCR targets using databases of clonotypes with defined specificities and disease associations. Although most of the clonotypes recognized unknown epitopes, we were able to identify a variety of CDR3 sequences associated with responses to pathogens, autoimmunity, allergy, and tumor surveillance. Of note is that our analysis included an annotation of all the CDR3 sequences described to date in the context of anti-CMV infection. Using this reference, we unveiled the dynamics and the impact of CMV serostatus and infection on diversity and pathological expansion across different patient groups. Although recent studies focused on posttransplant immune dysfunction after CMV reactivation (31, 21), here we reconstructed the anti-CMV–specific repertoire and evaluated its impact in the context of posttransplant immunity and across different patient risk categories. As highlighted by the study of CMV, the patterns of antigen-defined expansions followed organized trajectories, with the increased size of clonotypes associated with pathogens and tumor surveillance in later phases and inflation of autoreactive specificities in early stages, especially in the case of GVHD. Such dynamics may be essential in determining the development of either immunotolerance or alloreactive complications.

The specificity spectrum overlap analysis clarified the origin of the recipient repertoire; most of the clonotypes in recipients were newly generated or present at frequencies of less than 1/5000 (normalized depth), and only less than 3% were composed by clonotypes overlapping with the respective donor repertoire. Indeed, only a small fraction of pathologically expanded recipients’ specificities originated from expanded donors’ clonotypes, suggesting that either individual recipient’s antigenic drivers or very-low-frequency donor-derived clones expand in order to maintain the TCR complexity. These findings highlight the prevailing role of individual patients’ antigenic challenges and posttransplant thymic rebound in shaping the new TCR spectrum (7, 32).

Despite the limited sample size and the absence of cell sorting, which may help to better understand the relative contribution of CD4^+^ and CD8^+^ TCR repertoires on post–allo-HCT immune reconstitution and complications, our study provides a systematic and in-depth overview of TCRβ repertoire, taking into consideration the multidimensional nature of TCR diversification, expansion, and antigen recognition, together with the interactions dictated by posttransplant complications and pretransplant immunogenetic determinants.

Our analysis of post–allo-HCT TCR reconstitution demonstrates that NGS-based clonotyping of CDR3 tags can be used for analytic purposes to assess the status of T cell immune reconstitution as well as to detect and monitor specific T cell clones as markers of pathological immune processes.

## Methods

### DNA isolation.

Genomic DNA was isolated directly from fresh or cryopreserved unfractionated peripheral blood mononuclear cells with the Nuclei Lysis Solution (Promega) according to manufacturer’s instructions.

### TCRβ chain sequencing and analysis.

Immunosequencing of the CDR3 regions of human TCRβ chains was performed using the ImmunoSEQ Assay (Adaptive Biotechnologies), as previously described (33–35). In brief, extracted genomic DNA was amplified in a bias-controlled multiplex PCR, with a) a first PCR step consisting of forward and reverse amplification primers specific for every V and J gene segment to allow the amplification of the hypervariable CDR3 region and b) a second PCR adding a proprietary barcode sequence and Illumina adapter sequences. CDR3 libraries were sequenced on an Illumina MiSeq system according to the manufacturer’s instructions.

Rearrangement details from HCs (age matched with donors recruited in our cohort) derived from the Emerson and DeWitt study (data provided by the original publication and the ImmuneACCESS platform; refs. 36, 37). immunoSEQ Analyzer 3.0 suite (Adaptive Biotechnologies) was used for sample export and preliminary statistics and quality control steps while R Bioconductor (38) environment and Immunarch R (39) suite were used for all the downstream analyses (see Supplemental Methods).

All metrics were calculated based only on the “productive” rearrangements (translating a functional amino acid sequence, intended as templates that were in-frame and did not contain a stop codon in their sequence) within the normalized downsampled data set. Details concerning the bioanalytic workflow are reported in Supplemental Methods.

Sequencing results are accessible through the publicly available repository of the immuneACCESS platform (Adaptive Biotechnologies).

### Data sharing.

Genomic data that support the findings of this study have been deposited in the immuneACCESS platform (Adaptive Biotechnologies, https://clients.adaptivebiotech.com/pub/pagliuca-2021-jcii). All other remaining data (including rearrangement details of the downsampled TCR data sets) are available within the article and supplement.

### Statistics.

All metrics in the study were generally treated as continuous variables and categorized when needed. Median, IQR, mean, and 95% CIs were used where appropriate. Frequency and distribution of categorical variables were expressed as a percentage. For all relevant comparisons, after testing for normal distribution, comparative analyses between 2 groups were performed by 2-sided paired or unpaired Student’s *t* tests at a 95% CI. In case of not normally distributed data, Wilcoxon’s matched pairs signed-rank test at a 95% CI was used. Fisher’s exact test was applied for independent group comparisons, and in case of testing more than 2 groups, a 1-way ANOVA test was used. Cox regression and proportional hazard models for the competing risk subdistributions were used to assess the impact of diversity and overlap factor metrics on clinical outcomes (overall survival, cumulative incidence of relapse, and acute and chronic GVHD) in a univariable setting (40). OS was defined as the time from diagnosis to the last follow-up or death from any cause. Death at any time was used as competitive events for the cumulative incidence of relapse and chronic GVHD, while death before day 100 was used as competitive events for cumulative incidence of acute GVHD. For univariable analyses assessing the impact of factors influencing diversity, linear regression or generalized linear models were used where appropriate. *R*^2^ was used as a goodness-of-fit measure for the linear models. Binomial or Poisson’s distributions were used to assess the effect of binary or numeric predictors in the logistic models, respectively (41). All statistical tests were 2 sided, and a *P* < 0.05 was considered statistically significant. All of the analyses and data visualization were performed using the statistical computing environment R (4.0.0 R Core Team, R Foundation for Statistical Computing) and Excel (Microsoft 365).

### Study approval.

This is a prospective noninterventional monocentric study, conducted at our institution, under the institutional review board of the Cleveland Clinic Foundation (IRB 5024 and 4927). All consecutive patients undergoing a first allogeneic HCT for myeloid malignancies and who gave their consent to this research were included. Patient recruitment was conducted between September 2015 and January 2017; blood samples were collected prospectively from recipients (at –28 days for pretransplant and +30, +100, and +180 days after transplant) and respective related donors (in case of matched or haploidentical allo-HCT) for TCRβ sequencing (see Supplemental Figure 1, A and B).

All patients had been regularly followed until September 2020 (or death). Pertinent clinical data, including age, sex, disease diagnosis, type of transplant, HLA typing, conditioning regimen, GVHD prophylaxis, acute and chronic GVHD, disease relapse, and other complications, were collected. Objective medical data, including ancillary testing, laboratory results, medical complications, and medication profiles, were abstracted through standardized chart review after each visit. All patients provided written informed consent before enrollment, in accordance with the Helsinki Declaration of 1975, revised in 2008 (42).

## Author contributions

SP collected, analyzed, and interpreted the data; performed the bioinformatic and statistical analysis; and wrote the manuscript. CG and SH performed clinical data collection and participated in the analysis interpretation. RZ helped with statistical methodology development. SK, CMK, LT, MZ, AK, HA, BKJ, and YG helped in sample and data collection. TL and VV edited the manuscript, helped in data interpretation, and gave helpful intellectual insights during the study. HEC, BJP, BKH, and NSM actively participated in patient recruitment, management, and follow-up and critically revised the manuscript. JPM designed and conceptualized the study, interpreted the data analysis, and edited the manuscript. First and last authors took responsibility for the integrity and the accuracy of the data presented. All authors reviewed and approved the final version of this manuscript.

## Supplementary Material

Supplemental data

Supplemental table 1

Supplemental table 2

Supplemental table 3

Supplemental table 4

Supplemental table 5

Supplemental table 6

Supplemental table 7

Supplemental table 8

## Figures and Tables

**Table 1:**
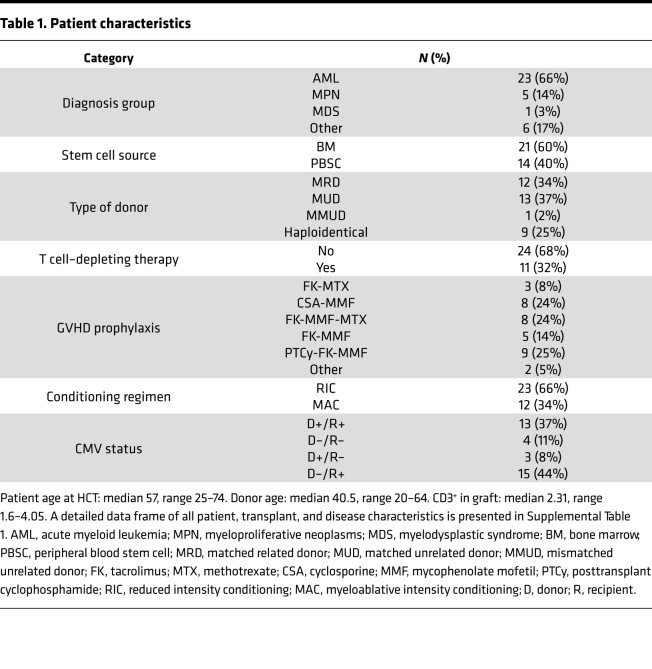
Patient characteristics

**Table 2:**
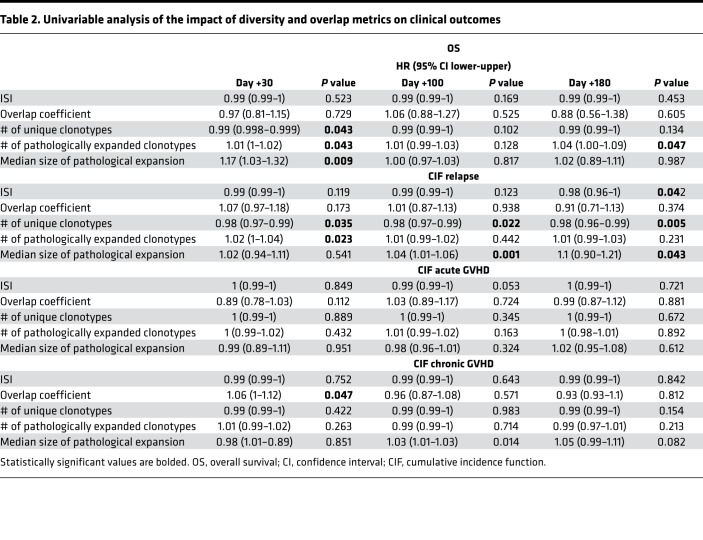
Univariable analysis of the impact of diversity and overlap metrics on clinical outcomes

## Version 1 07/08/2021

Electronic publication

## Version 2 07/13/2021

Updated supplemental files

## Version 3 08/03/2021

Updated supplemental tables
