# Supplementary material for: Clinical and basic implications of dynamic T cell receptor clonotyping in hematopoietic cell transplantation
Source: JCI Insight. 2021 Jul 8;6(13):e149080. doi: 10.1172/jci.insight.149080 (PMC8410023; doi:10.1172/jci.insight.149080)
Supplement: Supplemental data [file jciinsight-6-149080-s235.pdf]

SUPPLEMENTARY APPENDIX

Clinical and basic implications of dynamic T cell receptor clonotyping in hematopoietic cell transplantation

Simona Pagliuca[1,2], Carmelo Gurnari[1,3], Sanghee Hong[4], Ran Zhao[5], Sunisa Kongkiatkamon[1], Laila Terkawi[1], Misam Zawit[1], Yihong Guan[1], Hassan Awada[1], Ashwin Kishtagari[1], Cassandra M. Kerr[1], Thomas LaFramboise[6], Bhumika J. Patel[7], Babal Jha Kant[1], Hetty E. Carraway[7], Valeria Visconte[1], Navneet S. Majhail[4], Betty K. Hamilton[4], and Jaroslaw P. Maciejewski[1].

[1]Translational Hematology and Oncology Research Program, Department of Hematology and Oncology, Cleveland Clinic, Cleveland, OH
[2]University of Paris, Paris, France
[3]Department of Biomedicine and Prevention, PhD in Immunology, Molecular Medicine and Applied Biotechnology, University of Rome Tor Vergata, Rome, Italy
[4]Blood and Marrow Transplant Program, Department of Hematology and Oncology, Cleveland Clinic, Cleveland, Ohio
[5]Department of Quantitative Health Sciences, Cleveland Clinic, Cleveland, Ohio
[6]Department of Genetics and Genome Sciences, Case Western Reserve University, Cleveland, Ohio
[7]Leukemia program, Department of Hematology and Oncology, Cleveland Clinic, Cleveland, Ohio

Description of supplemental material:

All the supplementary tables are provided as separated Excel files.

Supplemental table 1: ST-1a: Terminology and metrics definition, ST-1b-c: Clinical annotations of patients and controls in study

Supplemental table 2: ST-2a: Quantitative characteristics of TCR repertoires of all the individual groups in study ST-1b: Rearrangement details of TCR repertoire in HC and donors (normalized dataset); ST-2c: Quantitative diversity metrics of TCR repertoires in HC and donors.

Supplemental table 3: ST-3a: Rearrangement details of shared clonotypes in donors ST-3b: Quantitation of pairwise clonotype sharing in donors.

Supplemental table 4: ST-4a: Rearrangement details of TCR repertoire in recipients; ST-4b: Quantitative diversity metrics in recipients; ST-4c: Distribution of clonotypes per size category; ST-4d: Distribution of clonotypes according to clonal expansion; ST-4e: Top clonal proportions; ST-4f: Quantitative characteristics of TCR repertoires of all the individual groups in study

Supplemental table 5: ST-5a: Rearrangement details of clonotypes shared between recipients and donors; ST-5b: Quantitation of shared and de novo clonotypes; ST-5c: Origin of shared clonotypes according to expansion status in donors; ST-5d: Pairwise overlap analysis between samples.

Supplemental table 6: ST-6a: Rearrangement details of samples at acute GVHD onset vs control group; ST-6b: Quantity and diversity metrics in GVHD vs non-GVHD samples; ST-6c: Rearrangement details of pre-GVHD and GVHD samples of index case CCF43.

Supplemental table 7: Rearrangement details of anti-CMV specificities

Supplemental table 8: Aggregated and curated dataset of known clonotype specificities.

Supplemental methods

Research strategy rationale

The present research seeks to investigate the dynamics of bulky T cell receptor (TCR) V beta repertoire reconstitution in patients receiving an allogeneic hematopoietic cell transplantation (allo-HCT) for myeloid malignancies. Pre-transplant and post-transplant blood specimens were collected at precise time points (-28, +30, +100 and +180 days). Quantitative characteristics of recipients' repertoire were compared with those of a control cohort of related donors for the patients enrolled in this study. A population of unrelated healthy controls was used for the baseline computation of normal diversity and overlap parameters and to define the strategy of categorization of clonal expansion patterns. We decided to analyze bulky TCR repertoires for different reasons:

-First, because of the inevitable lymphodepletion implicit in all transplant platforms and associated with the immunosuppressive procedures and the intensive chemotherapy-based pre-transplant treatments (standard practices in a population of patients with myeloid malignancies), a further breakdown would have decrease depth because of the cell sorting procedure.

-Second, the analysis of the clonotypic spectrum of complement determining region 3 (CDR3) related specificities sought to encompass specific T cell subtypes.

-Third, the design of this study was conceived to provide an easy-to-reproduce analytic framework helping our understanding of the superstructures involved in the overlap and diversity of TCR repertoires, reducing the level of their complexity to potentially translate this analytical strategy to other contexts and to clinical practice.

Sequencing platform accuracy

We decided to use for our study ImmunoSeq Assay (Adaptive Biotechnologies), a validated and scientifically accepted TCR sequencing platform. This assay utilizes a multiplex PCR-based assay, providing quantitative abundance data on either sorted or bulky cell populations (1).
Each immunoSEQ Assay contains rigorously designed synthetic immune templates as in-line controls plus optimized primers that ensure accurate, quantitative, and unbiased results with batch-to-batch

consistency. This platform takes advantage of a synthetic immune repertoire designed to represent all VJ-gene combinations. The measures against this synthetic repertoire enables to adjust the primer concentrations in order to significantly reduce amplification bias.

Sample quality and lymphocyte content

Per sample T cell content range was approximately 20-30% of the total cell population for each blood specimen and the starting DNA input concentration was 30-60 ng/µL, based on manufacturer's recommendations. Mean lymphocyte count was distributed as following: Pre: 1.3 10*9/l (IQR 0.88-1.67) Day + 30: 0.61 10*9/l (IQR 0.4-0.79); Day +100: 1.13 10*9/l (IQR 0.51-1.57); Day +180: 1.34 10*9/l (IQR 0.7-1.70).

Conditioning regimens and GvHD prophylaxis

High-resolution HLA-typing was used to select donors for allo-HCT. Donor types included matched related donor (MRD), matched (MUD) and mismatched unrelated donor (MMUD), and haploidentical donors. Stem cell sources were peripheral blood (PB), bone marrow (BM) while umbilical cord blood (UCB) was excluded for the small number of transplants performed with this graft source during the study period.
Myeloablative conditioning regimens (MAC) included busulfan (3.2 mg/kg/day for 4 days) combined with cyclophosphamide (60 mg/kg/day for 2 days) (2) or total body irradiation (TBI) of 1200 cGy combined with fludarabine (40 mg/m2/day for 4 days) (3). Reduced-intensity regimens (RIC) included fludarabine-based protocols, according to the disease and donor type. Standard protocols of immunosuppression including tacrolimus (FK) and short-term methotrexate (MTX) or cyclosporine (CsA) with mycophenolic mofetil (MMF) were used for GvHD prophylaxis for fully matched transplants. In addition, recipients of unrelated donor transplants received rabbit anti-thymocyte globulin (Thymoglobuline® 2.5 mg/kg/day for 2-4 days before transplantation) if indicated based on the underlying disease and the degree of donor HLA matching. Patients receiving an haploidentical allo-HCT received instead post-transplant cyclophosphamide (50mg/kg/day at day +3 and +4 after graft infusion) in combination with FK and MMF.

Bioanalytic workflow

Raw sequences were demultiplexed according to Adaptive's proprietary barcode sequences. Demultiplexed reads were then further processed to: remove adapter and primer sequences; identify and correct for technical errors introduced through PCR and sequencing. The data were filtered and clustered using a modified nearest-neighbor algorithm, to merge closely related sequences.

The resulting sequences were analyzed trough the ImmunoSeq Analizer 3.0 suite which allowed the annotation of the VDJ genes constituting each unique CDR3 and the translation of the encoded CDR3 amino acid sequence. V, D and J gene definitions were based on annotation in accordance with the IMGT database (www.imgt.org). A series of preliminary statistics were performed on non-normalized data (including *total templates*, *total productive templates*, number of *productive rearrangements*, *productive frequency, see Supp. table 1*).

After sample export (sample overview and rearrangement details), down-stream analyses were conducted with the Immunomind/immunarch v.0.6.6 R suite, and the R Bioconductor environment (4). To overcome the issue related to differences in intersample depth, we performed a normalization procedure, resampling the immune repertoire for all the specimens, to the smallest acceptable depth of the samples in study. More in details, the downsampling is an analytical strategy able to reduce in silico the sequencing depth and to enable instersample comparisons from imbalanced datasets, as, for their intrinsic nature, TCR repertoires are. Intrinsic drawbacks of this procedures concern the reduction of the sample size and the loss of samples with inadequate sample depth, however this strategy allow for comparisons of direct metrics (i.e. number of clonotypes, size of clonal expansion…) that would not be possible without a dataset normalization. Of note is that, when diversity of a repertoire is very high (i.e. in case of absence of expanded clonotypes and in presence of only non-expanded TCRs), very small frequency clones (i.e. # templates=1) may be affected by this procedure and may be lost after the resampling. This bias is relatively less important when the analytic plan consists in comparing diversity metrics (the richness of the sample in question would be higher regardless the low frequency clonotypes missing), however this issue becomes fundamental when the aim is to analyze the specificity spectrum, since a variety of non-expanded CDR3 sequences cannot be captured anymore for the qualitative analysis. Therefore, when appropriate we performed qualitative analyses (analysis of spectrum) on non-downsampled datasets.

The downsampling procedure was performed with the aforementioned tool using the following

parameters:

[*Immunarch function: repSample, parameters: .method = "downsample", .prob = TRUE*].

This choice allowed avoiding an important reduction of the sample size, while considering an adequate depth for the downstream analyses. For reference see https://github.com/immunomind/immunarch/blob/master/R/sampling.R.

Specifically, 105 samples were considered in the downsampled dataset (For distribution of samples across groups and time-points see **Fig.S2C**).

Several metrics were used to investigate HC and donor/recipient TCR clonal distribution profiles:

Diversity was characterized computing:

- The number of all unique clonotypes (richness)
- The size of each clonotypes (evenness or relative abundance).
- The Inverse Simpson`s index (a metrics derived from ecology and used to characterize the alpha diversity),(5),(6) calculated according to the following formula:

$$\frac{1}{\lambda} = \frac{1}{\sum_{i=1}^{R} p_i^2}$$

  where p_i is the proportional abundance for each clonotype and *R* is the total number of clonotypes in the sample.

Patterns of clonal expansion were defined as following:

- Non expanded: clonotypes present as singletons – 1 template – in the repertoire.
- Pathological expanded: clonotypes of size greater than the upper limit of the 95% confident interval (CI) of the clonal expansion distribution in HC and donors in the normalized dataset.
- Normal expanded: clonotypes of size ranging between >1 and the aforementioned threshold.

Similarity between repertoire was characterized as:

- direct number of shared clonotypes between sample pairs
- overlap coefficient which computes the proportion of overlap between two probability distributions, and is defined as the size of the intersection divided by the smaller of the size of

the two sets according to the following formula: (7)

$$oc(A,B) = \frac{|A \cap B|}{\min(|A|, |B|)}$$

Donor and recipient TCR repertoire in samples in study were annotated through the integration of a dataset aggregating all public databases supported by the Adaptive Immune Receptor Repertoire Community project (8). Briefly, after filtering for human TRB data, we downloaded the following public databasets: VDJDB (9) (https://vdjdb.cdr3.net/search), McPAS-TCR (10) (http://friedmanlab. weizmann.ac.il/McPAS-TCR/) and PIRD TBAdb (https://db.cngb.org/pird/tbadb/) (11). Immunarch dbAnnotate and trackClonotypes functions were used respectively to annotate and track TCR immune specificities. The comprehensive curated dataset of publicly known specificities is provided as Supplemental material (**Supp. Table 8**).

HED computation

HLA genotypes were obtained by NGS or sequence-specific oligonucleotide (PCR-SSO) -based methods.

Donor and recipient HED scores were computed starting from HLA genotypes, using the algorithm published by Pierini and Lenz, applying a customized perl script (https://sourceforge.net/projects/granthamdist/) for the calculation of the amino acid sequence divergence (12). Briefly, starting from a dictionary including all the protein sequences of exons 2 and 3 for class I alleles and exon 2 for class II alleles, assembled from the IPD-IMGT/HLA database, v.3.40 (13) we calculated HED for 6 class I (A, B, C) and II HLA loci (DRB1, DQB1, DPB1).

Means of class I and II were used as quantitative parameters for the univariable analysis of factors impacting TCR diversity.

References

1. Carlson CS, et al. Using synthetic templates to design an unbiased multiplex PCR assay. *Nature Communications* 2013;4(1). doi:10.1038/ncomms3680

2. Angelucci E, et al. Allogeneic bone marrow transplantation for hematological malignancies following therapy with high doses of busulphan and cyclophosphamide. *Haematologica* 1989;74(5):455–461.

3. Eder S, et al. Thiotepa-based conditioning versus total body irradiation as myeloablative conditioning prior to allogeneic stem cell transplantation for acute lymphoblastic leukemia: A matched-pair analysis from the Acute Leukemia Working Party of the European Society for Blood and Marrow Transplantation. *Am. J. Hematol.* 2017;92(10):997–1003.

4. Gentleman R. *Bioinformatics and computational biology solutions using R and Bioconductor*. New York: Springer Science+Business Media; 2005:

5. Kaplinsky J, Arnaout R. Robust estimates of overall immune-repertoire diversity from high-throughput measurements on samples. *Nature Communications* 2016;7(1). doi:10.1038/ncomms11881

6. Chiffelle J, et al. T-cell repertoire analysis and metrics of diversity and clonality. *Current Opinion in Biotechnology* 2020;65:284–295.

7. Inman HF, Bradley EL. The overlapping coefficient as a measure of agreement between probability distributions and point estimation of the overlap of two normal densities. *Communications in Statistics - Theory and Methods* 1989;18(10):3851–3874.

8. Rubelt F, et al. Adaptive Immune Receptor Repertoire Community recommendations for sharing immune-repertoire sequencing data. *Nat Immunol* 2017;18(12):1274–1278.

9. Shugay M, et al. VDJdb: a curated database of T-cell receptor sequences with known antigen specificity. *Nucleic Acids Res* 2018;46(D1):D419–D427.

10. Tickotsky N, et al. McPAS-TCR: a manually curated catalogue of pathology-associated T cell receptor sequences. *Bioinformatics* 2017;33(18):2924–2929.

11. Zhang W, et al. PIRD: Pan Immune Repertoire Database. *Bioinformatics* 2020;36(3):897–903.

12. Pierini F, Lenz TL. Divergent Allele Advantage at Human MHC Genes: Signatures of Past and Ongoing Selection. *Molecular Biology and Evolution* 2018;35(9):2145–2158.

13. Robinson J, et al. IPD-IMGT/HLA Database. *Nucleic Acids Research* 2019;gkz950.

Supplemental figures:

Figure S1: Study design. A: Description of the study design. B: Description of the immunosequencing and bioanalytic platform used for the characterization of the TCRβ repertoire. HSC: Hematopoietic stem cells; GvHD: Graft versus host disease; TCR: T cell receptor; CDR3: Complementary determining region 3

Figure S2: Characterization of the non-normalized repertoire. A: Total number of templates (total depth) across the sample groups. B: Total number of unique productive clonotypes. C: Clonotype size across donors and post-transplant samples. D: Sample size before and after the downsampling. Each colored

square indicates the presence of the sample at that given time-point. The number on the right represents the sum of the samples for each group.

Figure S3: Repertoire diversity in donors and recipient pre-transplant.
A: Number of unique clonotypes in donors and recipient pre-transplants (PRE). Violin plots. Mean donors (purple): 4748 (95%CI: 4366 -5128); median PRE (yellow): 4260 (95%CI:3928-4591). One dot per sample. (Donors: N=14, PRE: N= 25). Wilcoxon rank sum test (p=0.035).
B: Number of unique expanded clonotypes in donors and PRE. Violin plots. Mean donors (purple): 216 (95%CI: 135 -296); mean PRE (yellow): 255 (IQR:203-307). One dot per sample. (Donors: N=14, PRE: N= 25). Wilcoxon rank sum test (p=0.801)
C: Mean size of pathological expansion in donors and PRE. Violin plots. Mean size of expansion in donors: 14.6 (95%CI: 12 -17.1), mean PRE: 21 (I95%CI:18-25). One dot per sample.
D: Inverse Simpson index (ISI) distribution in donors (N=14) and PRE (N=25). Boxplots. Mean donors (purple): 2826 (95%CI 1726-3926); mean PRE (yellow): 1243 (95%CI: 704-1782). Wilcoxon rank sum test (p=0.005).
E: Average number per sample of pathologically expanded clonotypes according to clonal size category in PRE, expressed as fold change compared to donor group (see Supp. table 4 a,b and c). Of note, pre-transplant recipient samples have 5 times more hyperexpanded
specificities (clonal size >100 templates).

Figure S4: Clonal expansion of donor-recipient shared specificities. A: Linear correlation of clonal size of shared clonotypes in donors (Y-axis) and recipients (X-axis) at day +30 (green), +100 (red), +180 (light blue). Scatterplot in which each dot captures a clonotype. Overall, the expansion of the overlapping specificities in recipients correlated with the extent of expansion in donors.
B: Distribution of donor-recipient overlap coefficients among the post-transplant groups. Overall, the degree of overlap between recipients and donors remained constant across the time. For details of the calculation of this metrics see methods.

Figure S5: Clonal expansion of all known specificities. A: Distribution of the clonal sizes (intended as number of templates per clonotype) of clonotypes with known specificities in all the sample groups in study. Wilcoxon rank test. Only significant comparisons are shown in the figure. B: Distribution of the proportion of expanded clonotypes (# of templates >2) in all the sample groups in study. Analysis done on downsampled dataset.

Figure S6: I: **Distribution of VJ beta TCR families before and after GVHD in an index case.** Distribution of each V-J family in the total repertoire of pre-GVHD (upper panel) and GVHD (lower panel) samples from patient CCF43. 3D histogram illustrating the frequencies (Y-axis) of each TCR BV (X axis) and J (z-axis and histogram color) family. Top 15 clonotypes of most expanded families are reported as CDR3 aminoacidic strings, with character size proportional to the clonotype size.
Here the analysis was performed on the non-downsampled dataset, with specificity frequency calculated as the number of template of each unique clonotypes (clonal size)/total depth (total number of templates) *100.

Figure S7: Distribution of anti-CMV specificities in recipients
A: Number of anti-CMV clonotypes according to recipient and donor CMV serological status (N as in A). Violin plot. Recipient negative (pink dots): median 93 (IQR: 74-133); recipient positive (light blue dots): median 69 (IQR 59-75). Wilcoxon rank sum test: p=0.823. Donor negative (pink dots): median 78 (IQR: 68-109); donor positive: media 59 (35-89).

B: Frequency of anti-CMV specificities according to recipient and donor CMV serological status. Each dot represents a clonotype. Y-axis in log10 scale. Recipient negative: median 4.441e-05 (IQR: 3.262e-05 - 7.511e-05); recipient positive: median 3.995e-05 (IQR: 2.938e-05 - 7.969e-05). Wilcoxon rank sum test, p=0.124. Donor negative: median 6.091e-05 (IQR: 3.455e-05 - 8.191e-05); donor positive: median 3.262e-05 (IQR: 1.833e-05 - 6.030e-05). Wilcoxon rank sum test, p= <2e-16.
C: Number of anti-CMV specificities at day +100 according to incidence of CMV reactivation (Blood CMV DNAemia > 200 UI/ml) before day 100. Violin plot. Absence of CMV reactivation (Pink dots, N=20): median 71 (IQR: 60-91); presence of CMV reactivation (light blue dots, N= 9): median 79 (IQR 54-131). Wilcoxon rank sum test: p=0.721
D: Longitudinal analysis of impact of CMV reactivation on diversity metrics and dynamics of anti-CMV specificities in one index case (patient CCF33). After the viral reactivation the global diversity of the repertoire diminished in line with the post-transplant kinetics with a reduction of the total number of unique specificities, and an increase of the pathological expansion, along with the reduction in number and frequency (clonal size) of the anti-CMV specific clonotypes.

All the analyses including the study of anti-CMV CDR3 sequences were performed on the non-downsampled dataset in order to capture all the specificities (Supp. Table 7c). All the p here reported were two sided.

All the figures were created with or assembled on BioRender.com.

Figure S1

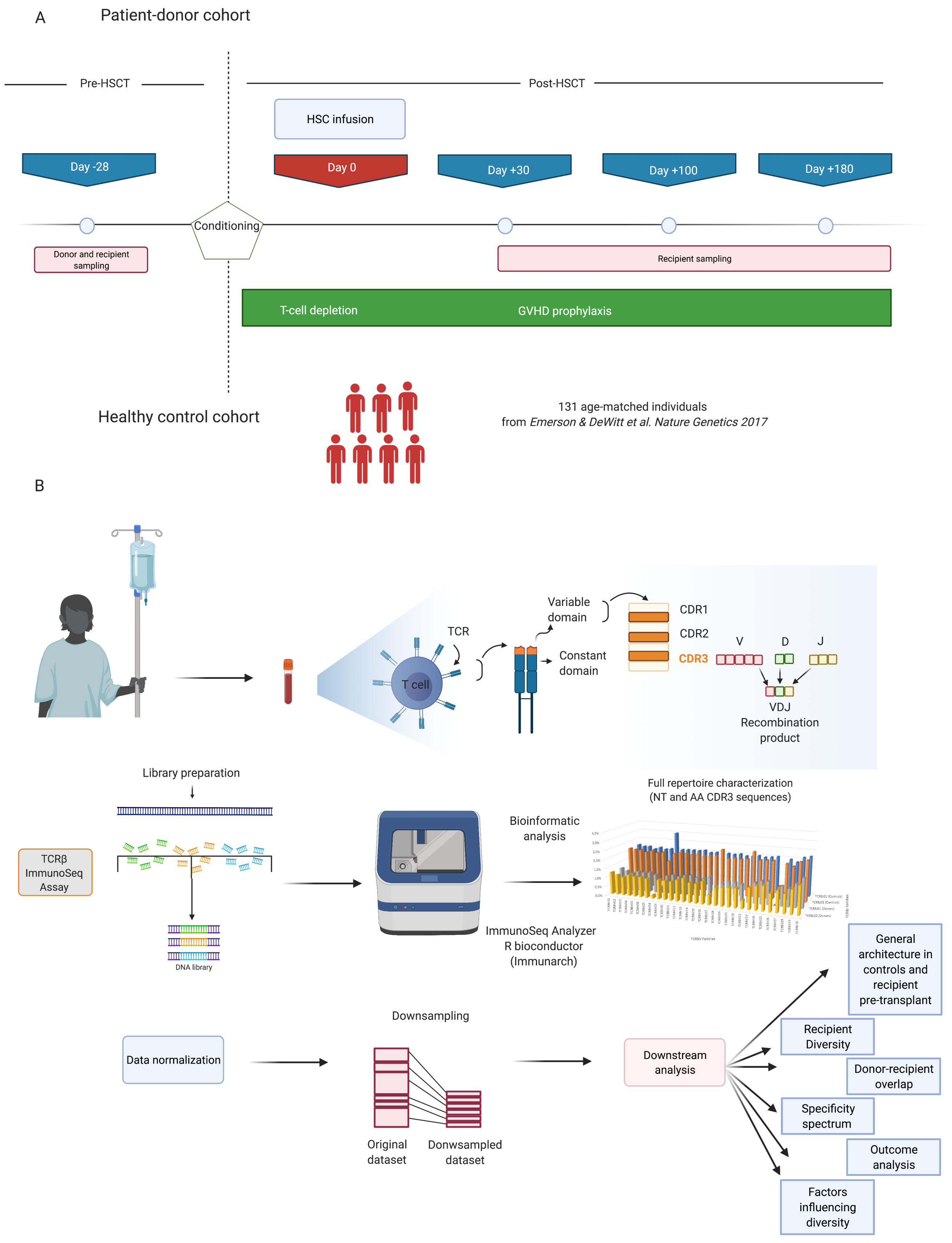
A
Patient-donor cohort
Pre-HSCT
Post-HSCT
HSC infusion
Day -28
Day 0
Day +30
Day +100
Day +180
Conditioning
Donor and recipient sampling
Recipient sampling
T-cell depletion
GVHD prophylaxis
Healthy control cohort
131 age-matched individuals
from *Emerson & DeWitt et al. Nature Genetics 2017*
B
TCR
T cell
Variable domain
Constant domain
CDR1
CDR2
CDR3
V
D
J
VDJ Recombination product
Library preparation
TCRβ ImmunoSeq Assay
DNA library
Bioinformatic analysis
ImmunoSeq Analyzer
R bioconductor
(Immunarch)
Full repertoire characterization
(NT and AA CDR3 sequences)
Data normalization
Downsampling
Original dataset
Donwsampled dataset
Downstream analysis
General architecture in controls and recipient pre-transplant
Recipient Diversity
Donor-recipient overlap
Specificity spectrum
Outcome analysis
Factors influencing diversity

Figure S2

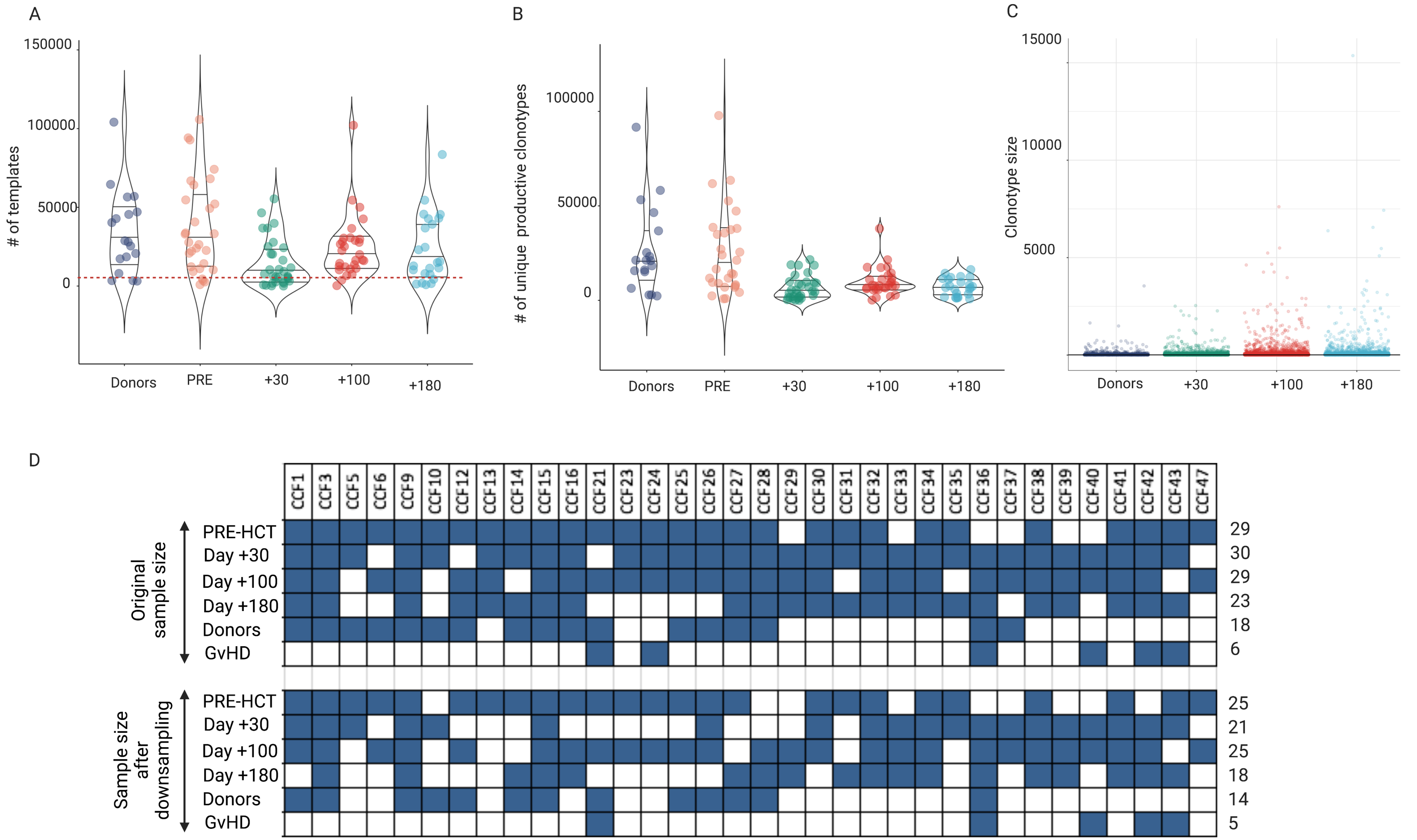
A
150000
100000
50000
0
# of templates
Donors
PRE
+30
+100
+180
B
100000
50000
0
# of unique productive clonotypes
Donors
PRE
+30
+100
+180
C
15000
10000
5000
Clonotype size
Donors
+30
+100
+180
D
CCF1 CCF3 CCF5 CCF6 CCF9 CCF10 CCF12 CCF13 CCF14 CCF15 CCF16 CCF21 CCF23 CCF24 CCF25 CCF26 CCF27 CCF28 CCF29 CCF30 CCF31 CCF32 CCF33 CCF34 CCF35 CCF36 CCF37 CCF38 CCF39 CCF40 CCF41 CCF42 CCF43 CCF47
Original sample size
PRE-HCT 29
Day +30 30
Day +100 29
Day +180 23
Donors 18
GvHD 6
Sample size after downsampling
PRE-HCT 25
Day +30 21
Day +100 25
Day +180 18
Donors 14
GvHD 5

Figure S3

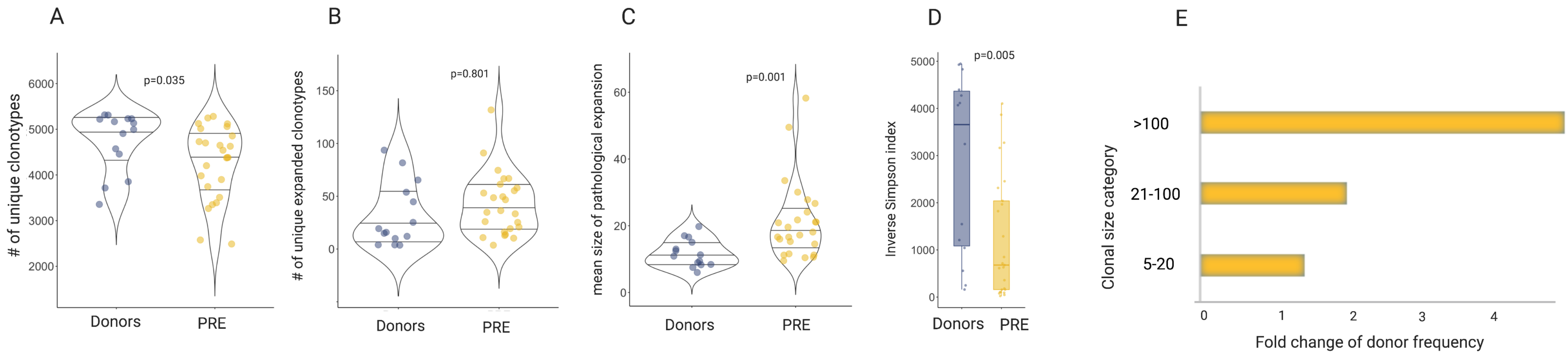
A
# of unique clonotypes
6000
5000
4000
3000
2000
p=0.035
Donors
PRE
B
# of unique expanded clonotypes
150
100
50
0
p=0.801
Donors
PRE
C
mean size of pathological expansion
60
40
20
0
p=0.001
Donors
PRE
D
Inverse Simpson index
5000
4000
3000
2000
1000
0
p=0.005
Donors
PRE
E
Clonal size category
>100
21-100
5-20
0
1
2
3
4
Fold change of donor frequency

Figure S4

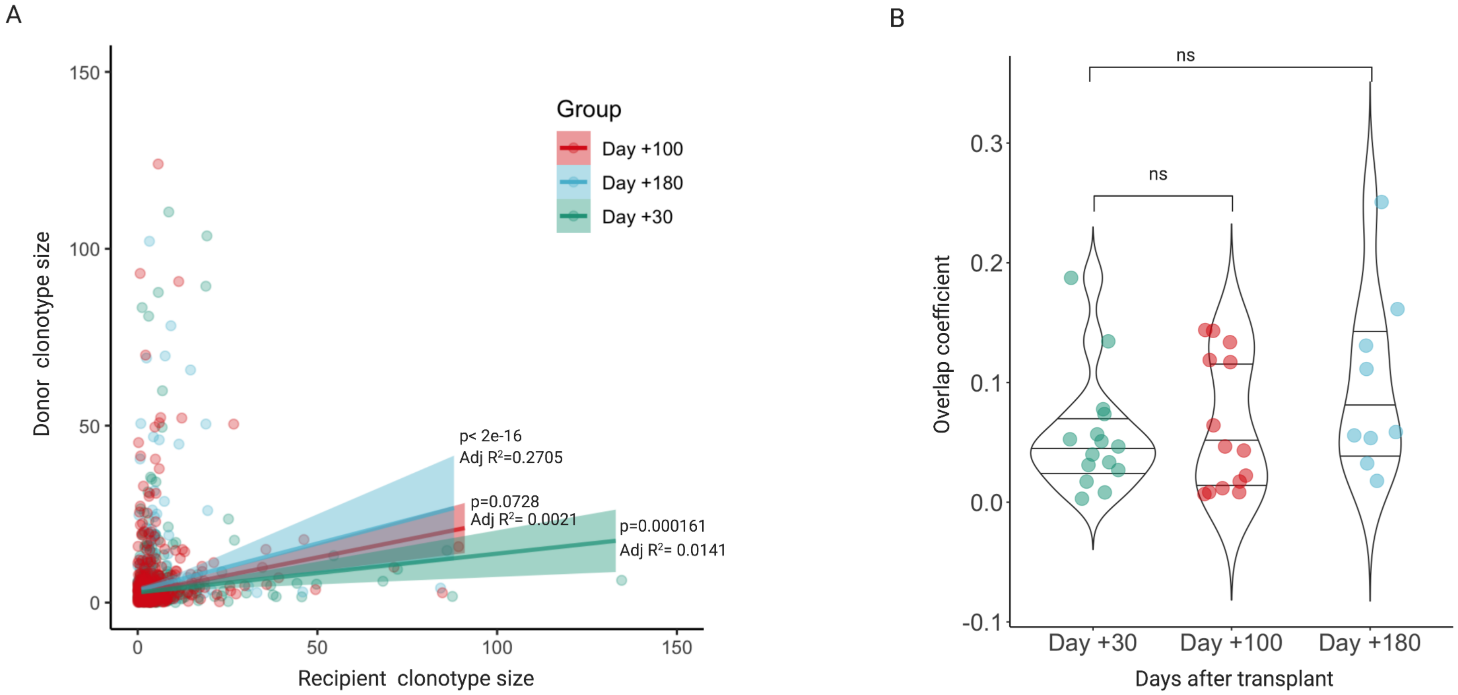
A
Group
Day +100
Day +180
Day +30
Donor clonotype size
Recipient clonotype size
p< 2e-16
Adj R²=0.2705
p=0.0728
Adj R²= 0.0021
p=0.000161
Adj R²= 0.0141
B
ns
ns
Overlap coefficient
Day +30
Day +100
Day +180
Days after transplant

Figure S5

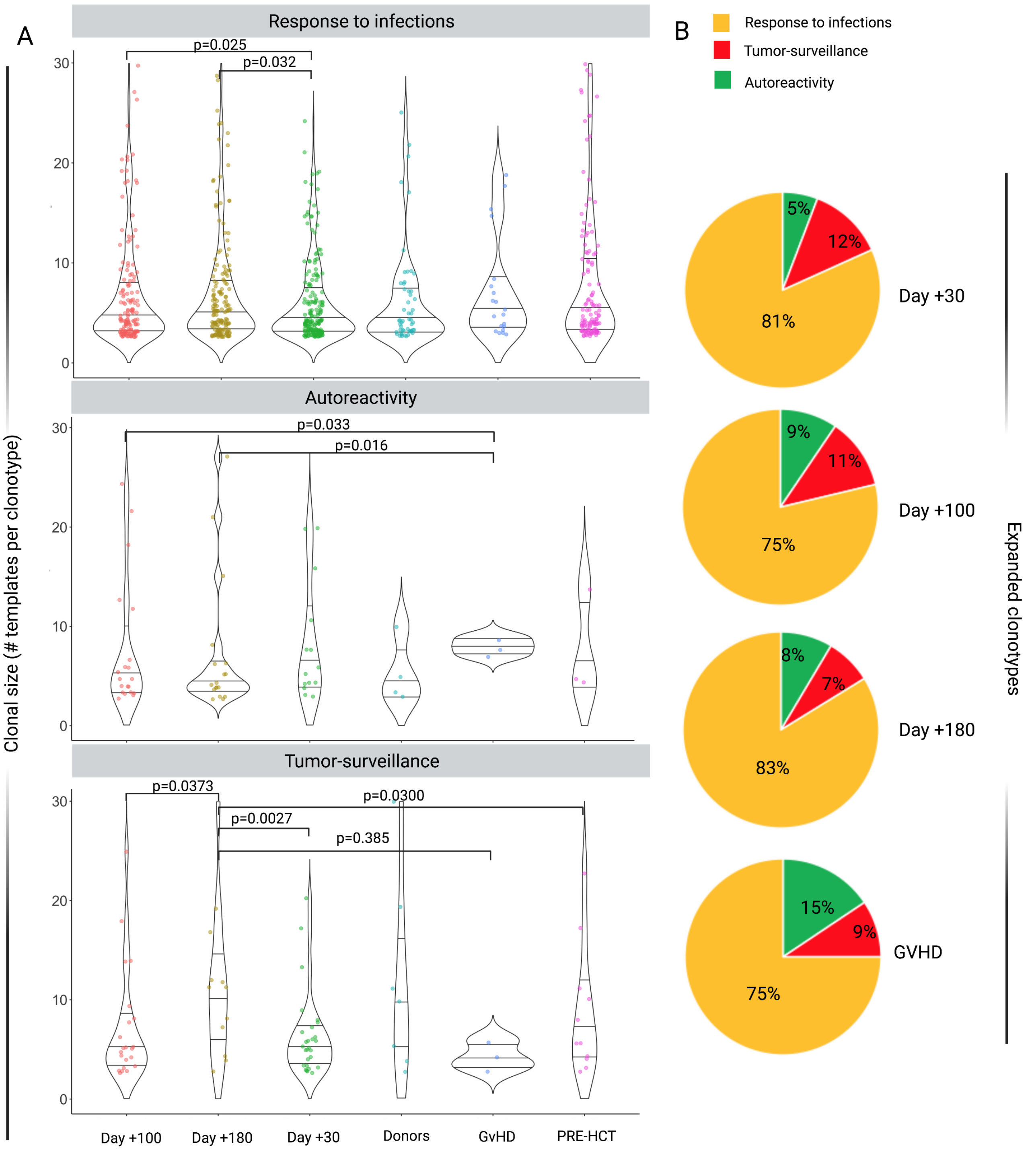
A
Response to infections
p=0.025
p=0.032
Autoreactivity
p=0.033
p=0.016
Tumor-surveillance
p=0.0373
p=0.0300
p=0.0027
p=0.385
Clonal size (# templates per clonotype)
Day +100
Day +180
Day +30
Donors
GvHD
PRE-HCT
B
Response to infections
Tumor-surveillance
Autoreactivity
5%
12%
81%
Day +30
9%
11%
75%
Day +100
8%
7%
83%
Day +180
15%
9%
75%
GVHD
Expanded clonotypes

Figure S6

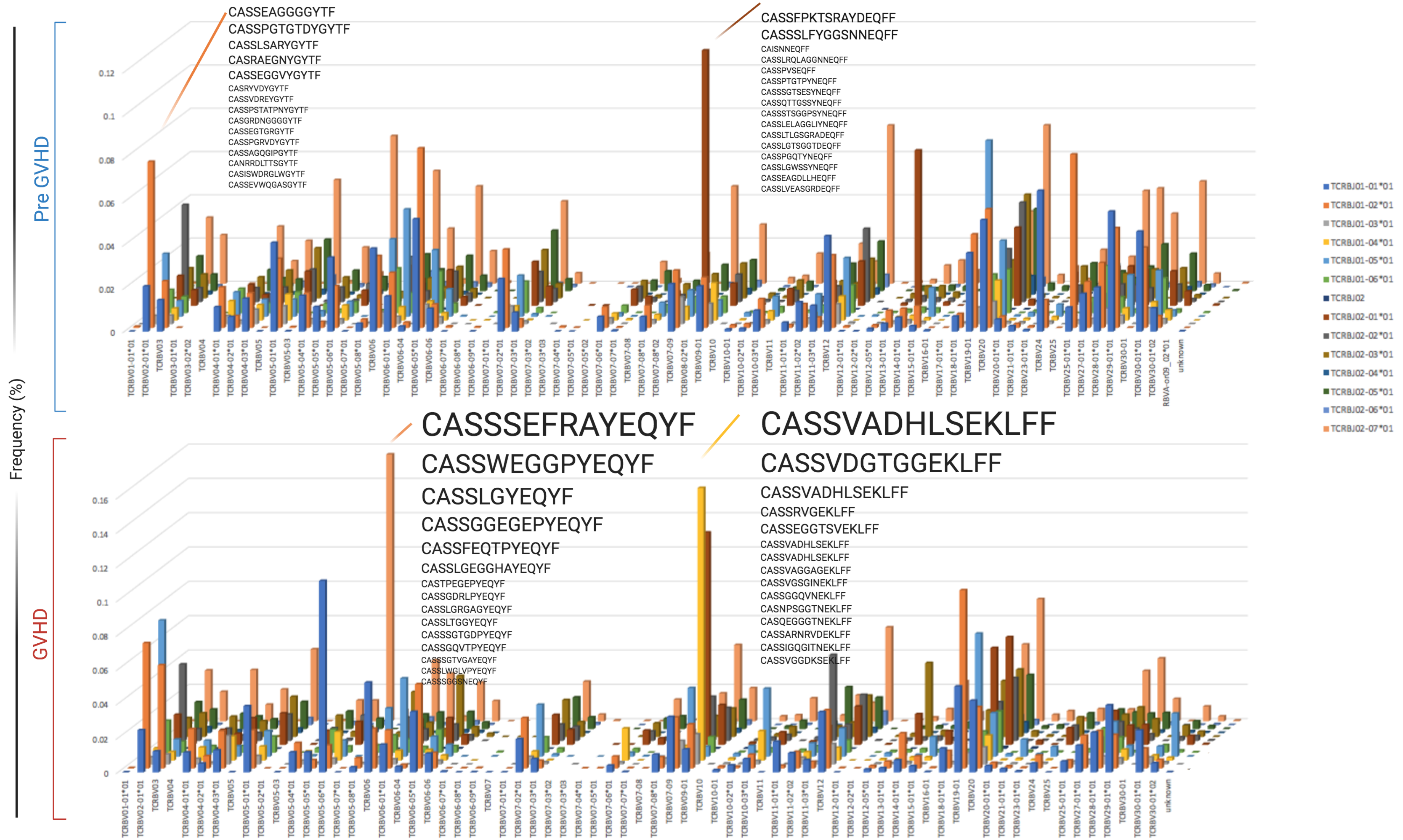
Frequency (%)
Pre GVHD
GVHD
CASSEAGGGGYTF
CASSPGTGTDYGYTF
CASSLSARYGYTF
CASRAEGNYGYTF
CASSEGGVYGYTF
CASRYVDYGYTF
CASSVDREYGYTF
CASSPSTATPNYGYTF
CASGRDNGGGGYTF
CASSEGTGRGYTF
CASSPGRVDYGYTF
CASSAGQGIPGYTF
CANRRDLTTSGYTF
CASISWDRGLWGYTF
CASSEVWQGASGYTF
CASSFPKTSRAYDEQFF
CASSSLFYGGSNNEQFF
CAISNNEQFF
CASSLRQLAGGNNEQFF
CASSPVSEQFF
CASSPTGTPYNEQFF
CASSSGTSESYNEQFF
CASSQTTGSSYNEQFF
CASSSTSGGPSYNEQFF
CASSLELAGGLIYNEQFF
CASSLTLGSGRADEQFF
CASSLGTSGGTDEQFF
CASSPGQTYNEQFF
CASSLGWSSYNEQFF
CASSEAGDLLHEQFF
CASSLVEASGRDEQFF
CASSSEFRAYEQYF
CASSWEGGPYEQYF
CASSLGYEQYF
CASSGGEGEPYEQYF
CASSFEQTPYEQYF
CASSLGEGGHAYEQYF
CASTPEGEPYEQYF
CASSGDRLPYEQYF
CASSLGRGAGYEQYF
CASSLTGGYEQYF
CASSSGTGDPYEQYF
CASSGQVTPYEQYF
CASSGTVGAYEQYF
CASSLWGLVPYEQYF
CASSGGSNEQYF
CASSVADHLSEKLFF
CASSVDGTGGEKLFF
CASSVADHLSEKLFF
CASSRVGEKLFF
CASSEGGTSVEKLFF
CASSVADHLSEKLFF
CASSVADHLSEKLFF
CASSVAGGAGEKLFF
CASSVGSGINEKLFF
CASSGGQVNEKLFF
CASNPSGGTNEKLFF
CASQEGGGTNEKLFF
CASSARNRVDEKLFF
CASSIGQGITNEKLFF
CASSVGGDKSEKLFF
0.12
0.1
0.08
0.06
0.04
0.02
0
0.16
0.14
0.12
0.1
0.08
0.06
0.04
0.02
0
TCRBJ01-01*01
TCRBJ01-02*01
TCRBJ01-03*01
TCRBJ01-04*01
TCRBJ01-05*01
TCRBJ01-06*01
TCRBJ02
TCRBJ02-01*01
TCRBJ02-02*01
TCRBJ02-03*01
TCRBJ02-04*01
TCRBJ02-05*01
TCRBJ02-06*01
TCRBJ02-07*01

Figure S7

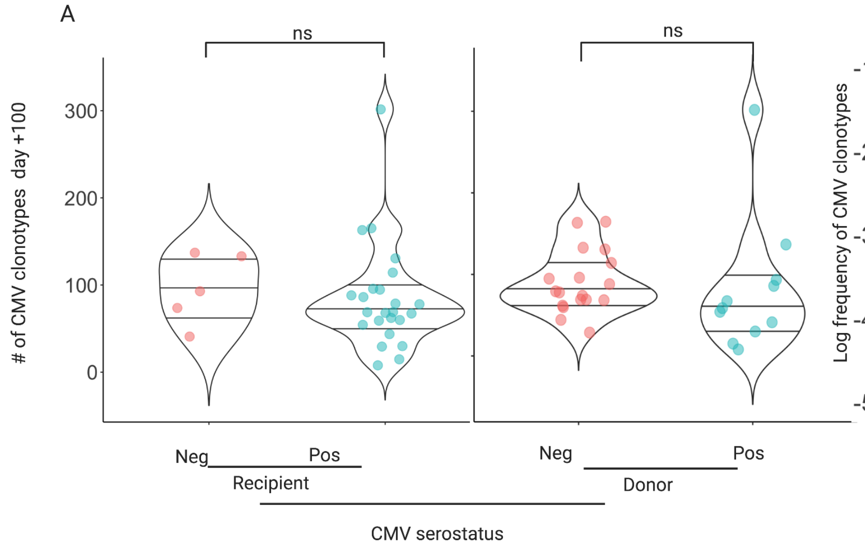
A
ns
ns
# of CMV clonotypes day +100
300
200
100
0
Neg
Pos
Recipient
Neg
Pos
Donor
CMV serostatus

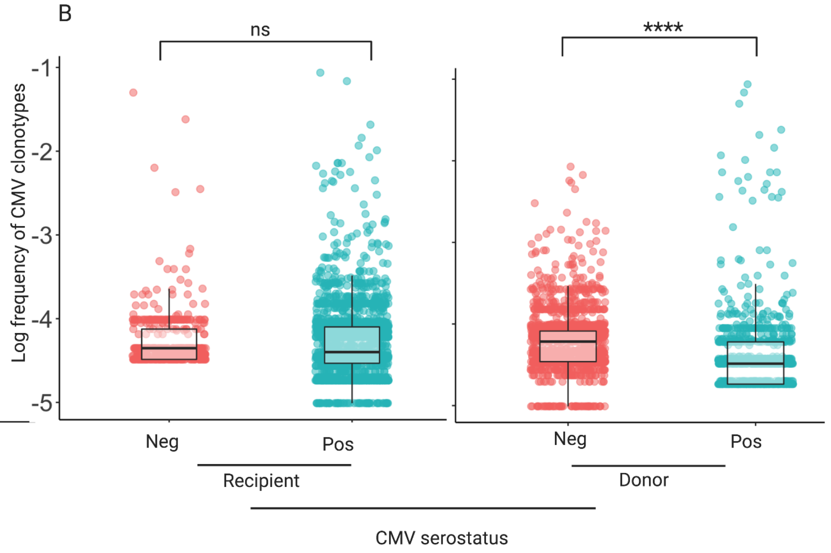
B
ns
****
Log frequency of CMV clonotypes
-1
-2
-3
-4
-5
Neg
Pos
Recipient
Neg
Pos
Donor
CMV serostatus

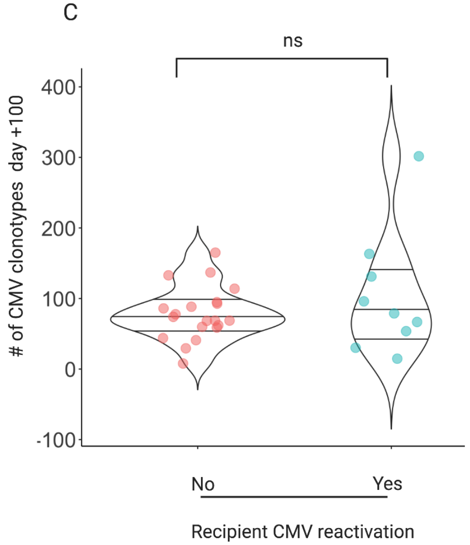
C
ns
# of CMV clonotypes day +100
400
300
200
100
0
-100
No
Yes
Recipient CMV reactivation

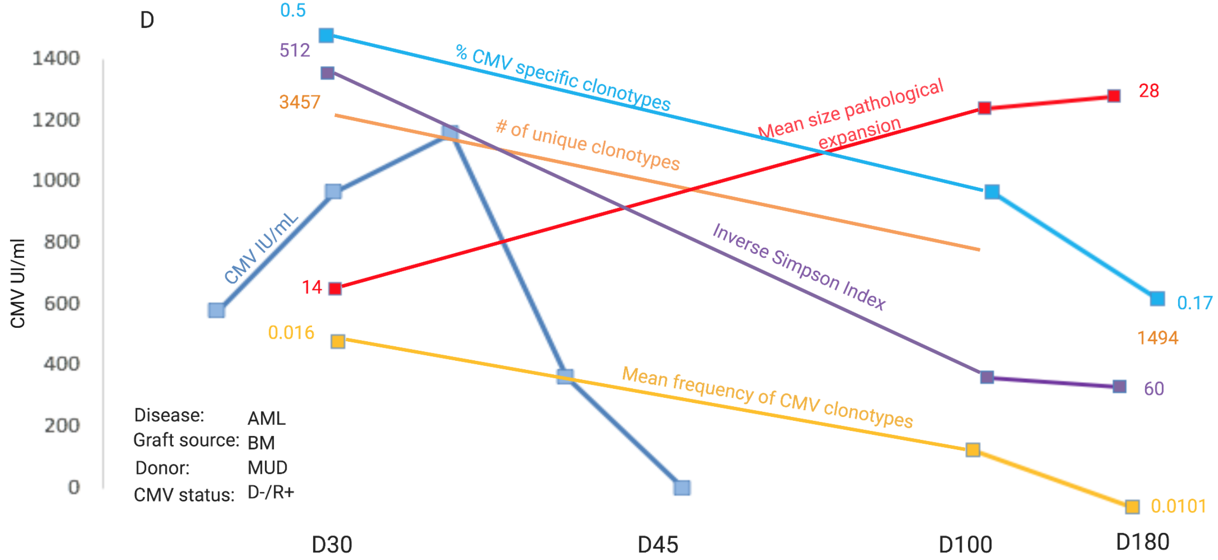
D
CMV UI/ml
1400
1200
1000
800
600
400
200
0
0.5
512
3457
% CMV specific clonotypes
# of unique clonotypes
Mean size pathological expansion
28
CMV IU/mL
14
Inverse Simpson Index
0.17
1494
0.016
Mean frequency of CMV clonotypes
60
0.0101
Disease: AML
Graft source: BM
Donor: MUD
CMV status: D-/R+
D30
D45
D100
D180

Supplemental Table 1a: Terminology and metrics definition

**Rearrangement**	Nucleotide sequence resulting from the recombination process and encoding for a given V (D) J chain. It includes the CDR3 sequence. It is productive if it is in frame and does not contain stop codons, encoding for a fully functional aminoacidic sequence
**Template**	Each productive rearrangement contained in the repertoire
**Clonotype**	A TCR with a given specificity. It is identified through the unique nucleotide rearrangements and CDR3 sequences. A variable number of identical templates may be present (as product of the T-cell activation and proliferation), defining its clonal expansion
**Clonal size**	Defined either as number of templates in a given clonotype, or as frequency (number of templates/total depth). Characterizes the relative abundance of each clonotype.
**Depth**	Total number of rearrangements in a repertoire (productive and non-productive)
**Downsampling**	Statistical strategy used to model unbalanced data (TCR repertoires with different depths). It subsets all the classes (samples' depths) in the dataset (repertoires from different samples) so that their class frequencies match the least prevalent class (repertoire with lower depth).
**Clonal expansion**	Defines all the clonotypes of size greater than 1 template
**Pathological clonal expansion**	Arbitrarily defined in this study as an expansion greater than the upper limit of the 95% CI found in healthy subjects. This threshold may variate with the downsampling strategy used to model the dataset. It is proposed as internal criteria to be used to describe the characteristics of repertoires with different sample depths.
**Number of unique clonotypes**	Defines the richness, one of the components of the diversity
**Number of unique expanded clonotypes**	Number of all clonotypes of size >1 template
**Number of pathologically expanded clonotypes**	Number of all clonotypes of size >the 95%CI upper limit found in controls
**Diversity of the repertoire**	Defined as number of all the constituents (clonotypes) of the repertoire (richness) and their relative abundance (clonal size or frequency, also known as evenness).

Sample tags for use on Adaptive Biotechnologies ImmuneACCESS platform

Patient ID	Pre-HCT	Day +30	Day +100	Day +180	Donors	GVHD
CCF1	14913	15218	15431	15710	15023	
CCF3	15216	15480	15670	15943	15226	
CCF5	15243	15463			15250	
CCF6	15323		15691		15275	
CCF9	15472	15622	15844	16113	15503	
CCF10	15598	15757			15611	
CCF12	15801		16181	16506	15828	
CCF13	15825	15970	16175	16470		
CCF14	15847	15987		16503	15853	
CCF15	15888	16023	16309	16546	15878	
CCF16	15924	16093	16439	16608	15925	
CCF21	16144		16536		16160	16654
CCF23	16168	16411	16687			
CCF24	16348	16444	16636			16460
CCF25	16358	16458	16662		16393	
CCF26	16143	16436	16652		16174	
CCF27	16423	16544	16773	17046	16445	
CCF28	16446	16547	16768	17041	16454	
CCF29		15240	15433	15718		
CCF30	15363	15719	15913	16162		
CCF31	15519	15646		16097		
CCF32	15597	15754	15969	16158		
CCF33		15865	16068	16415		
CCF34	15807	15928	16135	16447		
CCF35	15910	16062		16551		
CCF36		16081	16364	16588	15971	16679
CCF37		16079	16350		15967	
CCF38	15990	16127	16422	16626		
CCF39		16311	16469	16744		
CCF40		16410	16575			16410
CCF41	16394	16502	16726	16999		
CCF42	16441	16578	16797	17059		16769
CCF43	16525	16655		17186		17017
CCF47	16710		17058			
CCF48	16709		17092		16741	
